# Supplementary material for: Discovery of plant chemical defence mediated by a two-component system involving β-glucosidase in Panax species
Source: Nat Commun. 2024 Jan 18;15:602. doi: 10.1038/s41467-024-44854-7 (PMC10796634; doi:10.1038/s41467-024-44854-7)
Supplement: Supplementary file 1 — Supplementary Information [file 41467_2024_44854_MOESM1_ESM.pdf]

# Supplementary Materials

## Discovery of plant chemical defence mediated by a two-component system involving β-glucosidase in *Panax* species

Li-Juan Ma[1,9], Xiao Liu[2,9], Liwei Guo[3], Yuan Luo[2], Beibei Zhang[2,4], Xiaoxue Cui[2], Kuan Yang[3], Jing Cai[5], Fang Liu[1], Ni Ma[6], Feng-Qing Yang[7], Xiahong He[3,8,*], She-Po Shi[2,*], Jian-Bo Wan[1,*]

[1] State Key Laboratory of Quality Research in Chinese Medicine, Institute of Chinese Medical Sciences, University of Macau, Macao, China

[2] Modern Research Center for Traditional Chinese Medicine, Beijing University of Chinese Medicine, Beijing, China

[3] State Key Laboratory for Conservation and Utilization of Bio-Resources in Yunnan, Yunnan Agricultural University, Kunming, Yunnan, China

[4] State Key Laboratory of Bioactive Substance and Function of Natural Medicines, Institute of Materia Medica, Chinese Academy of Medical Sciences and Peking Union Medical College, Beijing 100050, China

[5] School of Ecology and Environment, Northwestern Polytechnical University, Xi'an, Shaanxi, China

[6] Department of Product Development, Wenshan Sanqi Institute of Science and Technology, Wenshan University, Wenshan, Yunnan, China

[7] Department of Pharmaceutical Engineering, School of Chemistry and Chemical Engineering, Chongqing University, Chongqing 401331, China.

[8] Ministry of Education Key Laboratory for Forest Resources Conservation and Utilization in the Southwest Mountains of China, Southwest Forestry University, Kunming, Yunnan 650224, China

[9] These authors contributed equally: Li-Juan Ma, Xiao Liu

* Correspondences: jbwan@um.edu.mo (J.W); shishepo@163.com (S.S); hxh@swfu.edu.cn (X.H.)

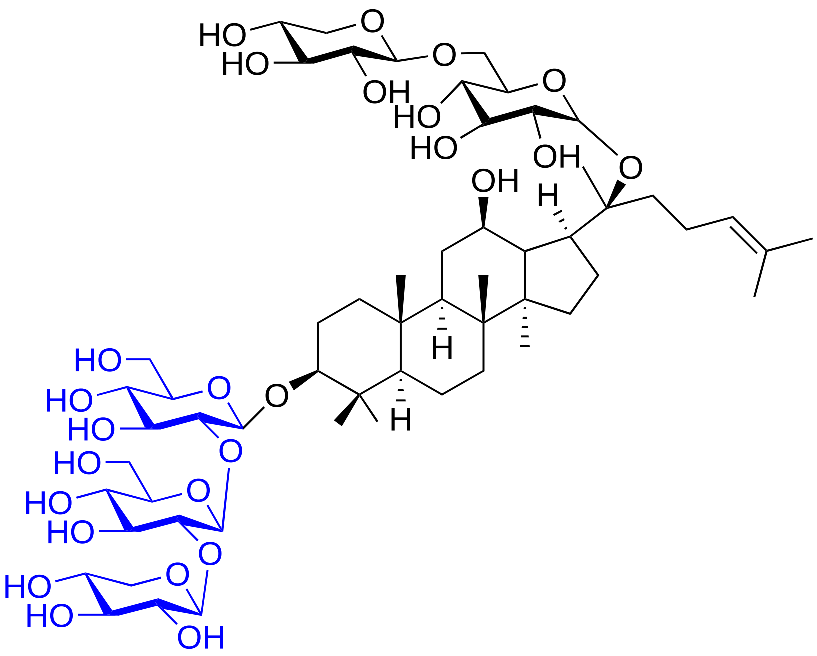


**Supplementary Fig. 1** Chemical structure of notoginsenoside Fc (**5**).

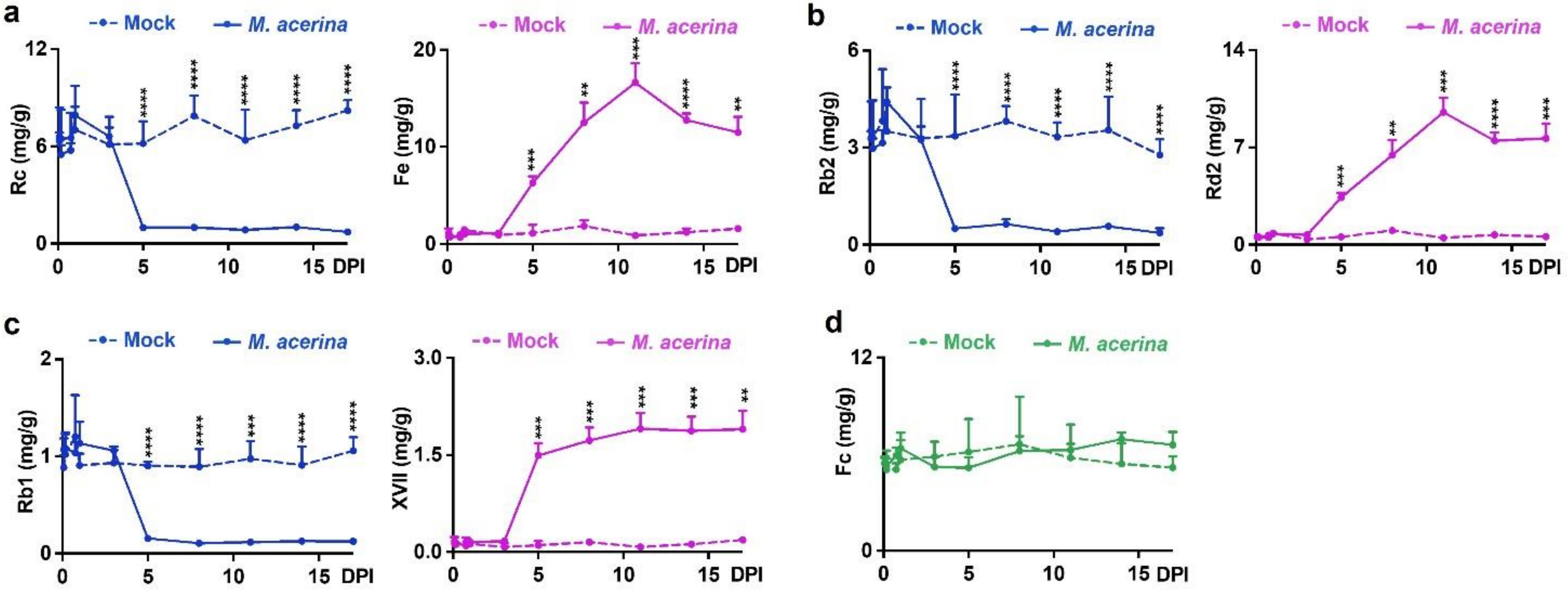


**Supplementary Fig. 2** Absolute contents of Rc/Fe (**a**) Rb2/Rd2 (**b**), Rb1/XVII (**c**), and Fc (**d**) in decayed PNL (Zone I) during a time-course post the inoculation with *M. acerina*. n=4 and 5 biologically independent samples for mock and *M. acerina*-inoculated groups, respectively. The data are expressed as mean ± SEM. Statistical significance was calculated using Student's *t*-test. ***, $p < 0.001$; ****, $p < 0.0001$. Source data are provided as a Source Data file.

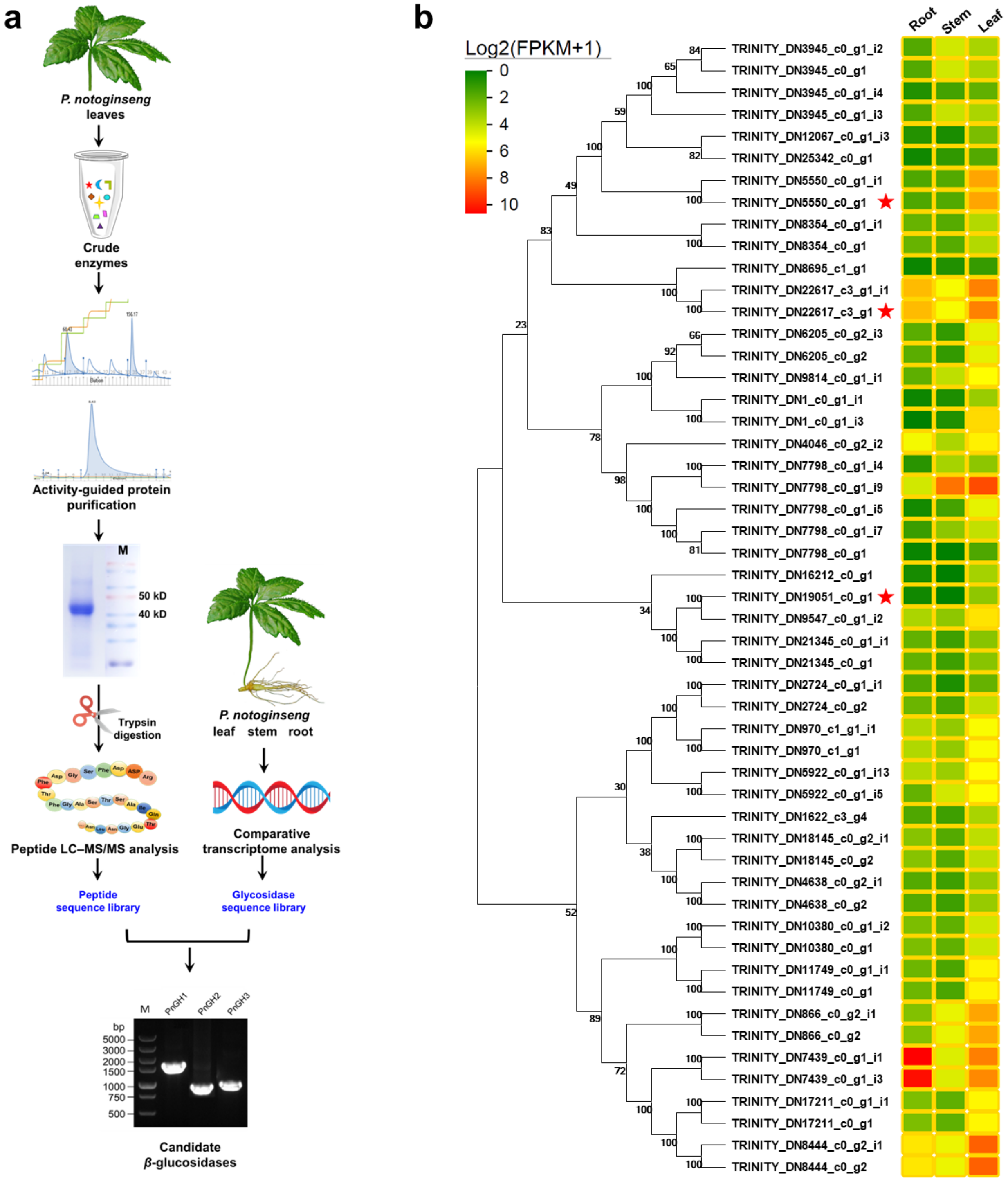


**Supplementary Fig. 3** (**a**) *β*-glucosidase exploration from *P. notoginseng* based on activity-guided protein purification and comparative transcriptome analysis. (**b**) Transcripts and unigenes annotated as glycosidase and their expression levels in different *P. notoginseng* tissues. Sequences with a SEQUEST HT score greater than 10 were labeled with stars.

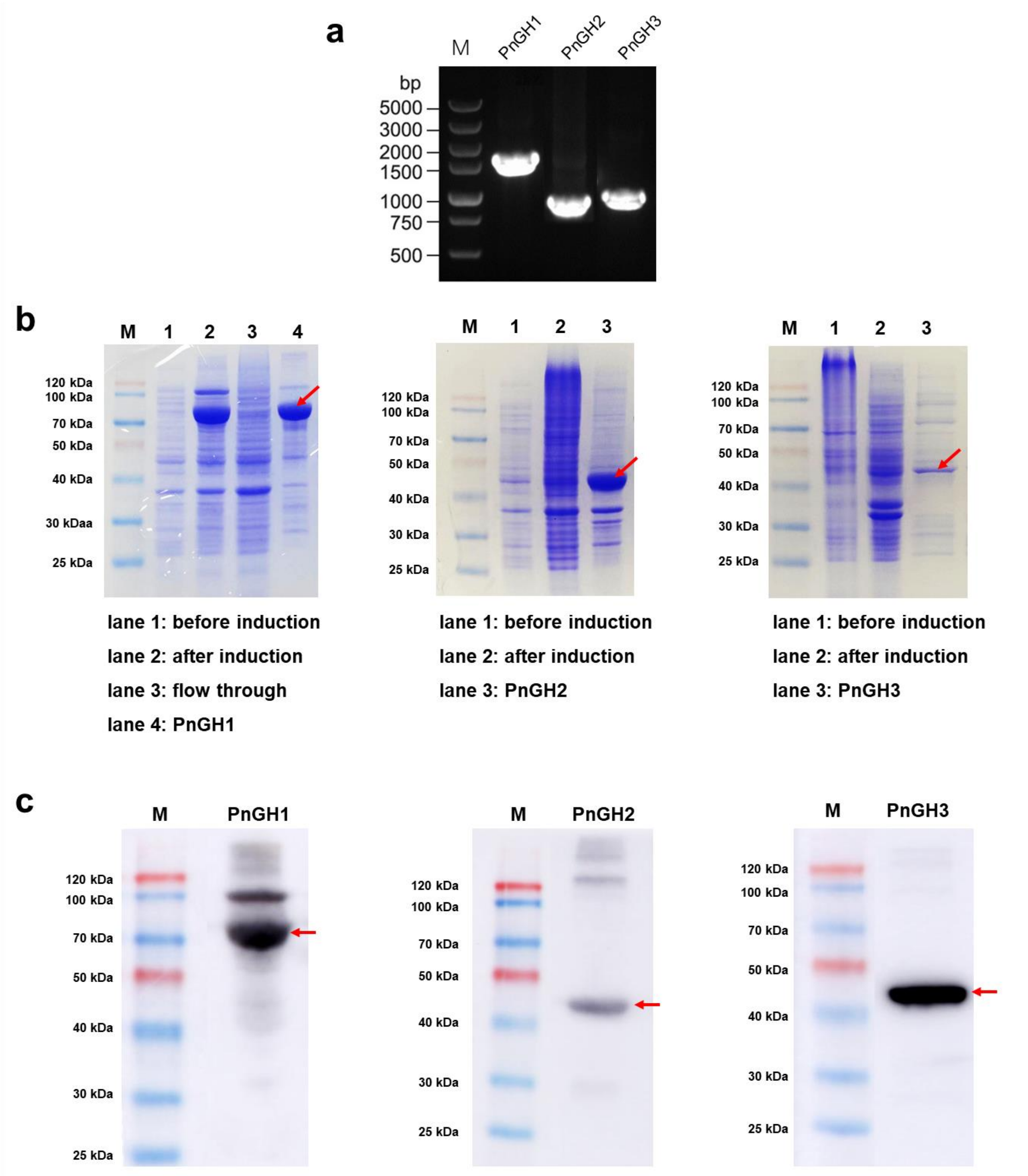


**Supplementary Fig. 4** (**a**) Gene cloning of *PnGH1−3* genes from *P. notoginseng*. (**b**) Heterogenous expression of $His_6$-tag fusion proteins of PnGH1−3 in *E. coli*. (**c**) Western blot analysis of PnGH1−3 proteins with anti-His-tag mAb antibody. The experiment was repeated independently three times with similar results.

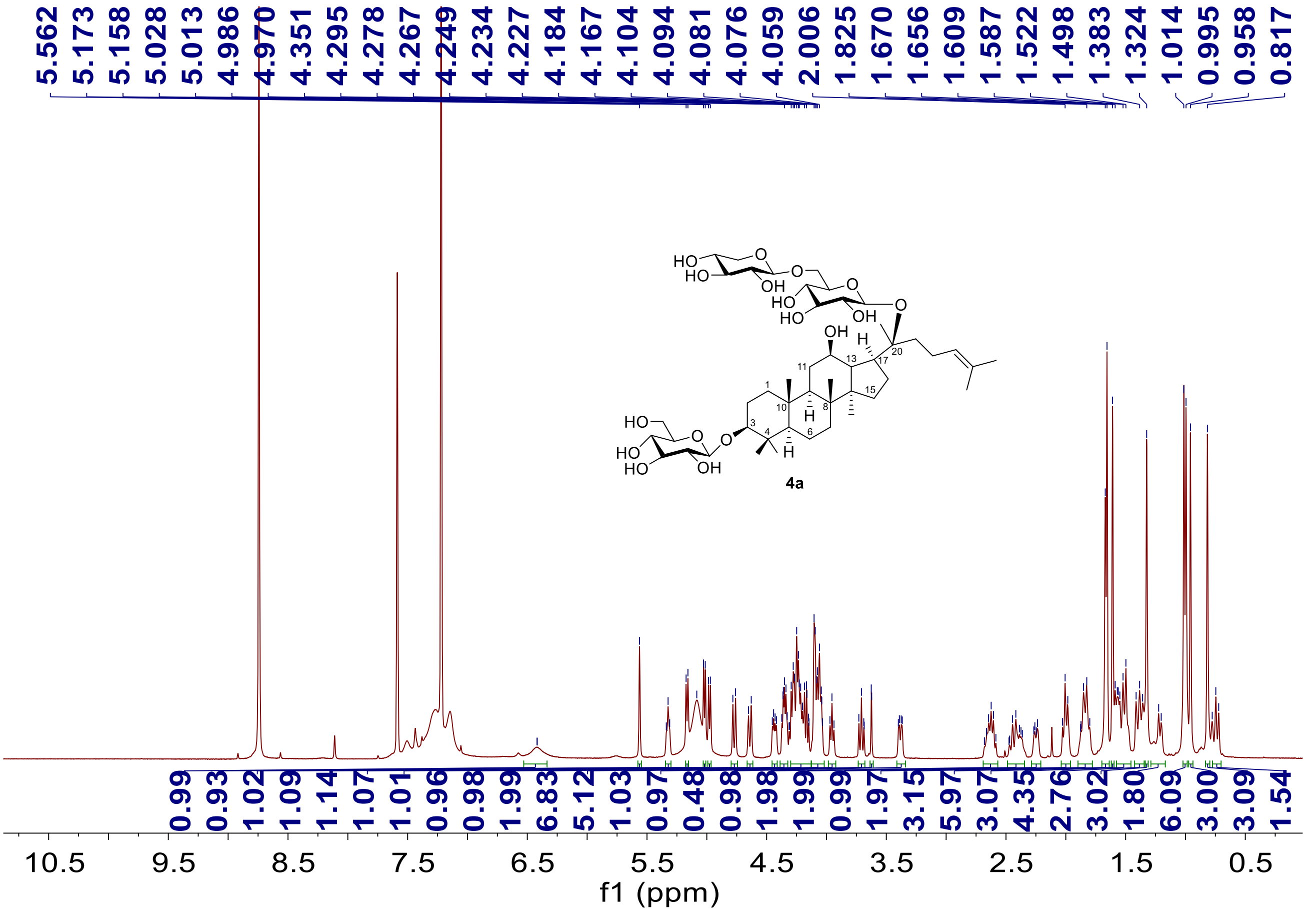


**Supplementary Fig. 5** $^{1}$H NMR spectrum (500 MHz) of product **4a** in pyridine-*d*$_5$. Notoginsenoside Fd (**4a**), HRESI-MS (negative) *m/z* [M + HCOO]$^-$ 961.5378 (calcd. 961.5378 for $C_{48}H_{81}O_{19}$); $^{1}$H NMR (500 MHz, pyridine-*d*$_5$): $\delta_H$ 5.33 (1H, t, *J* = 7.0 Hz, H-24), 5.17 (1H, d, *J* = 7.5 Hz, H-glc-1''), 5.02 (1H, d, *J* = 7.5 Hz, H-xyl-1'''), 4.98 (1H, d, *J* = 8.0 Hz, H-glc-1'), 4.78 (1H, br.d, *J* = 11.0 Hz, H-glc-6''a), 4.65 (1H, br.d, *J* = 11.5 Hz, H-glc-6'a), 4.43 (1H, dd, *J* = 11.5, 5.5 Hz, H-glc-6'b), 4.36 (2H, overlapped, H-glc-6''b and H-xyl-6'''b), 3.95 (1H, t, *J* = 8.5 Hz, H-glc-2''), 3.71 (1H, t, *J* = 11.5 Hz, H-xyl-6'''a), 3.39 (1H, dd, *J* = 12.0, 4.0 Hz, H-3), 1.67 (3H, s), 1.66 (3H, s), 1.61 (3H, s), 1.32 (3H, s), 1.01 (3H, s), 0.99 (3H, s), 0.96 (3H, s), 0.82 (3H, s), other protons were not assigned due to highly overlapped.

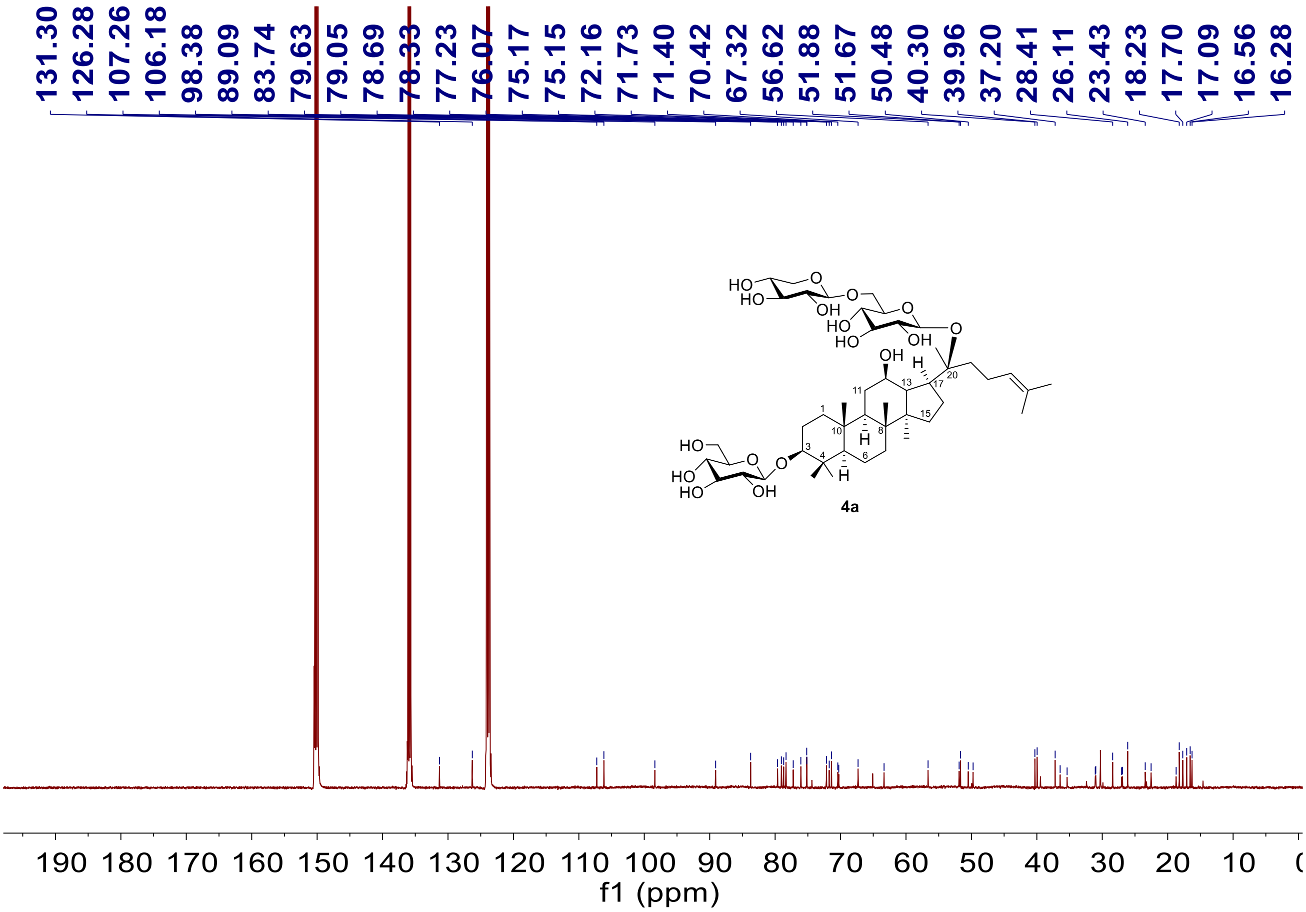


**Supplementary Fig. 6** $^{13}$C NMR spectrum (125 MHz) of product **4a** in pyridine-$d_5$. Notoginsenoside Fd (**4a**), $^{13}$C NMR (125 MHz, pyridine-$d_5$): $\delta_C$ 131.3, 126.3, 107.3, 106.2, 98.4, 89.1, 83.7, 79.6, 79.1, 78.7, 78.3, 77.2, 76.1, 75.2, 75.1, 72.2, 71.7, 71.4, 70.4, 70.3, 67.3, 63.4, 56.6, 51.9, 51.7, 50.5, 49.7, 40.3, 39.9, 39.4, 37.2, 36.4, 35.4, 31.1, 31.0, 28.4, 27.1, 26.9, 26.1, 23.4, 22.5, 18.7, 18.2, 17.7, 17.1, 16.6, 16.3.

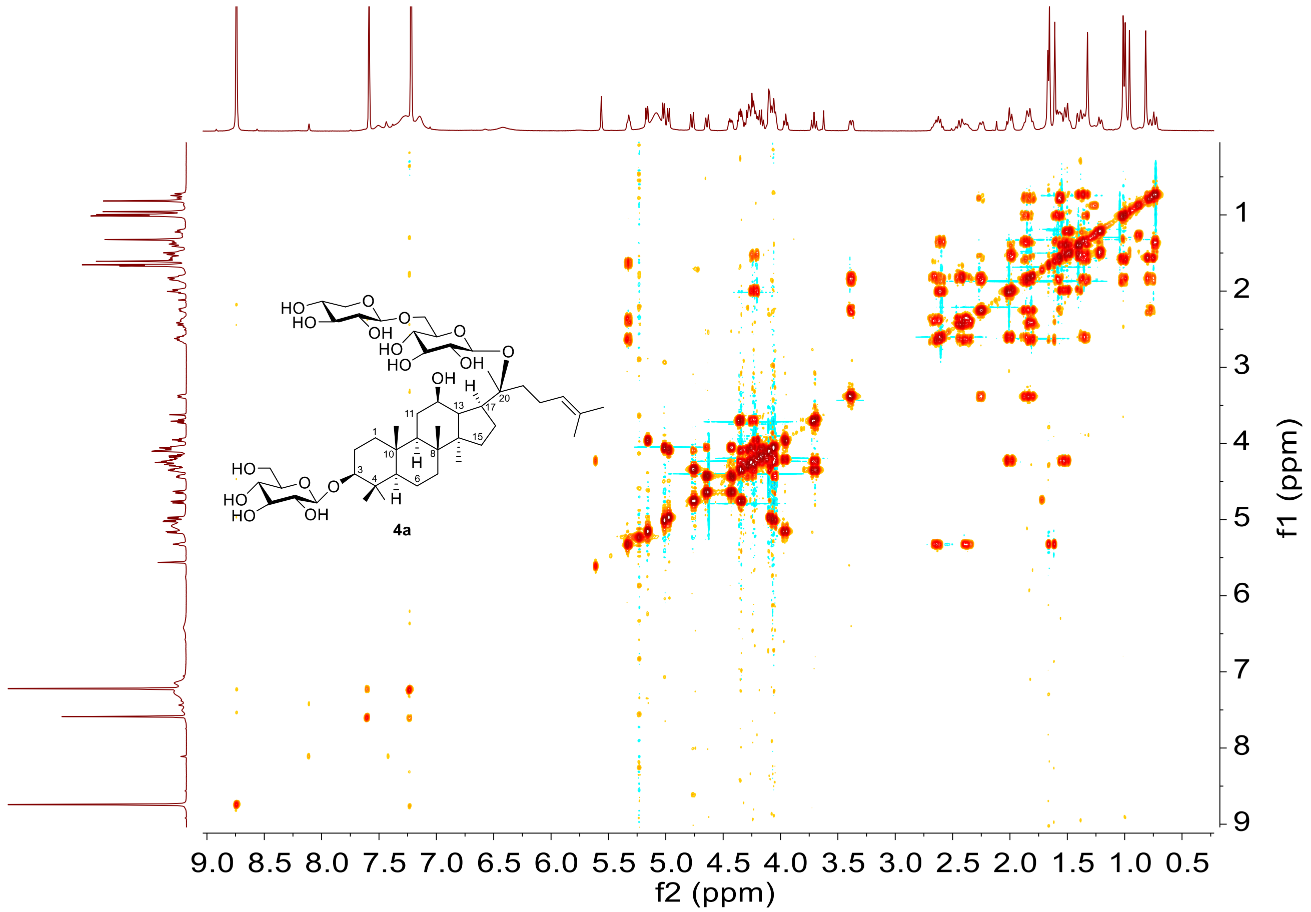


**Supplementary Fig. 7** $^{1}$H-$^{1}$H COSY spectrum of product **4a** in pyridine-$d_5$

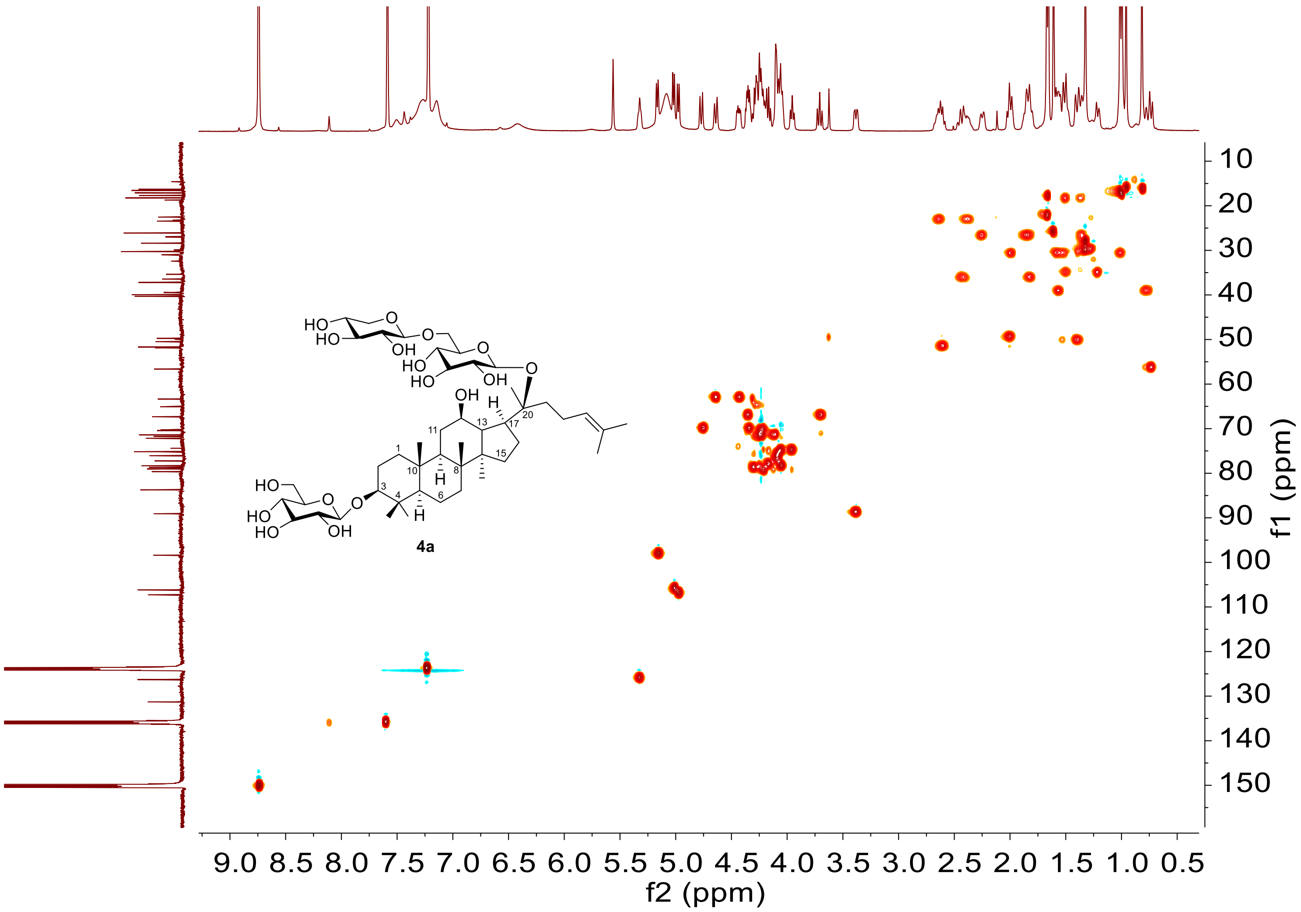


**Supplementary Fig. 8** HSQC spectrum of product **4a** in pyridine-$d_5$

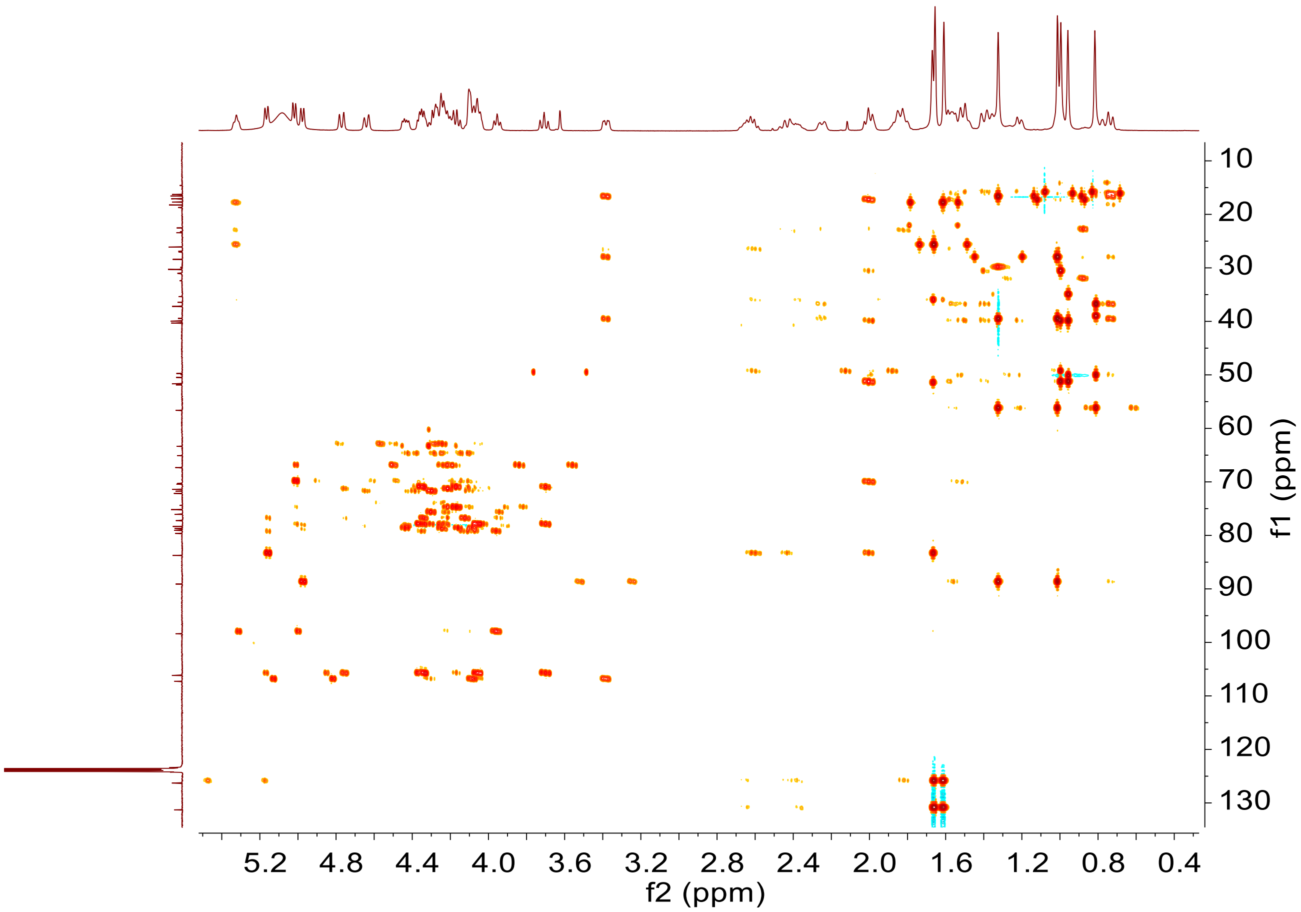


**Supplementary Fig. 9** HMBC spectrum of product **4a** in pyridine-$d_5$

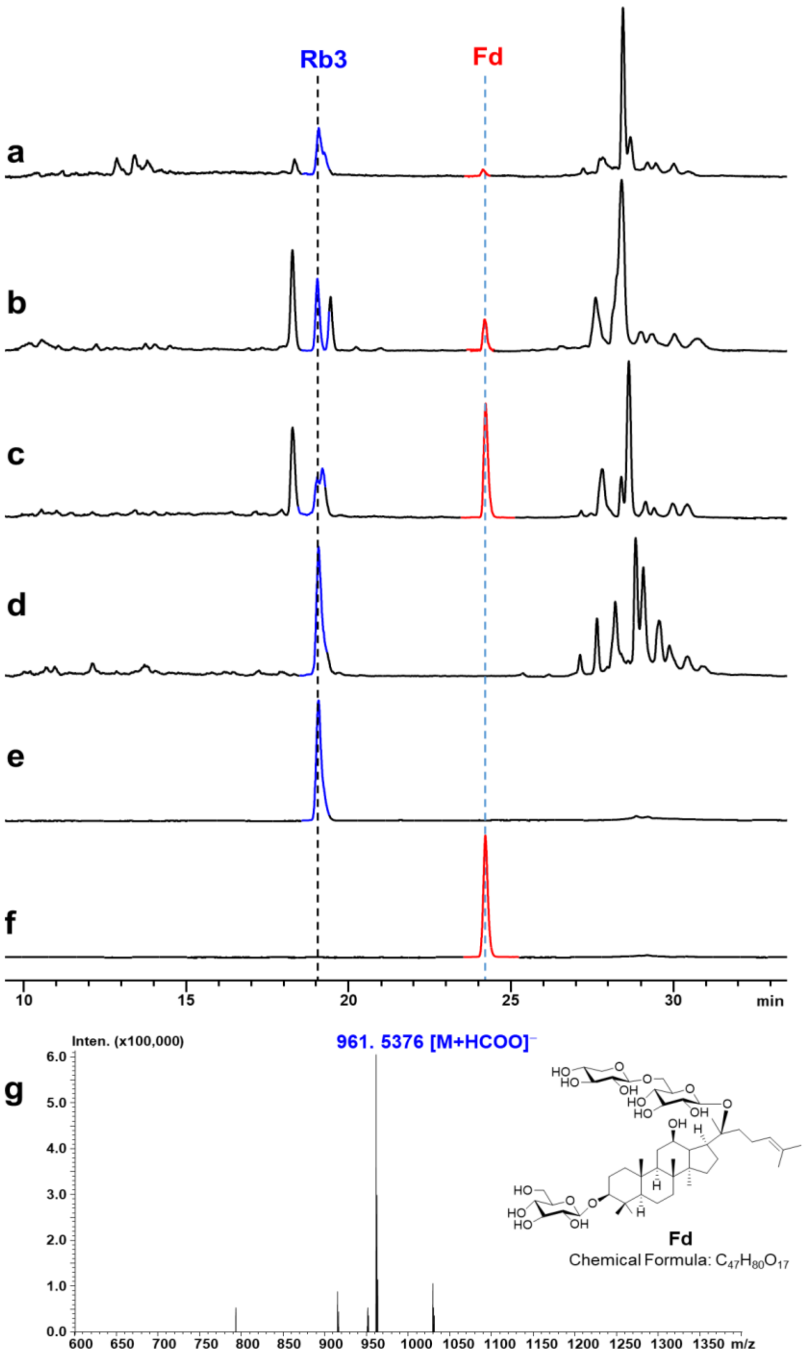


| Formula (M) | Ion | Meas. m/z | Pred. m/z | Df. (mDa) | Df. (ppm) | Iso | DBE |
|---|---|---|---|---|---|---|---|
| C47 H80 O17 | [M+HCOO]- | 961.5376 | 961.5378 | -0.2 | -0.21 | 0.00 | 8.0 |

**Supplementary Fig. 10** Transient expression of *PnGH1* in *N. benthamiana*. (**a-f**) HPLC-ELSD spectra of *N. benthamiana* leaves after agroinfiltration under different treats. *N. benthamiana* leaves were infiltrated with *Agrobacterium* harboring pCAMBIA1300-35S-*PnGH1* for 48 h. Then 200 μM Rb3 was injected. The leaves were harvested after 2h (a) and 48 h (b), respectively. (**c**) Co-HPLC analysis of sample b and Fd standard. (**d**) Leaves infiltrated with *Agrobacterium* harboring pCAMBIA1300-35S vector were used as control. (**e**) Rb3 standard. (**f**) Fd standard. (**g**) HRESI MS spectrum of the product peak (Fd) in *N. benthamiana* leaves infiltrated with *Agrobacterium* harboring pCAMBIA1300-35s-*PnGH1*.

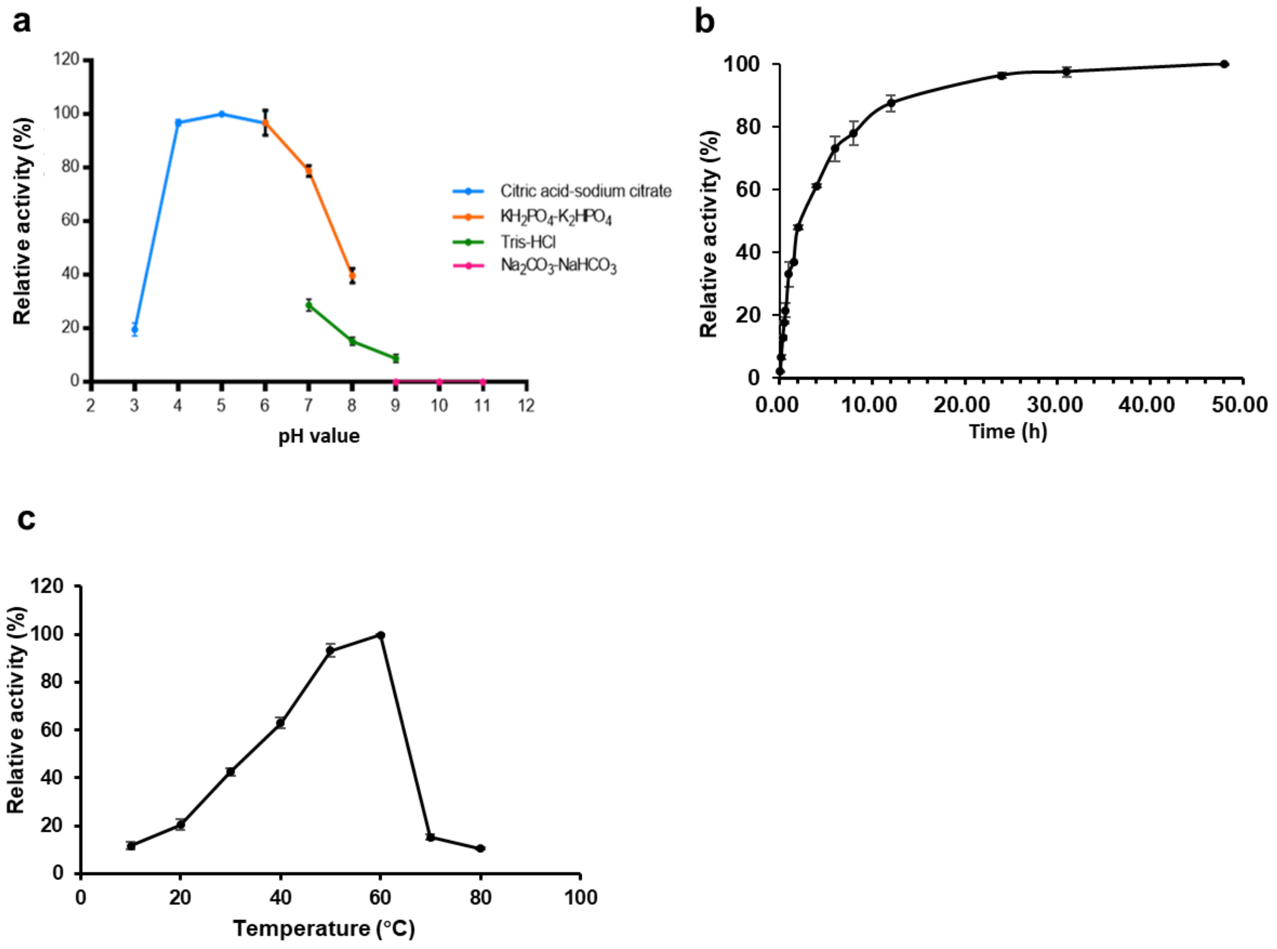


**Supplementary Fig. 11** Effects of reaction buffer pH (**a**), reaction time (**b**), and temperature (**c**) on the activities of PnGH1. All assays were carried out in three parallel using Rb3 as the substrate. Data are presented as mean ± SD. n=3 biologically independent samples. The source data are provided in Source Data file.

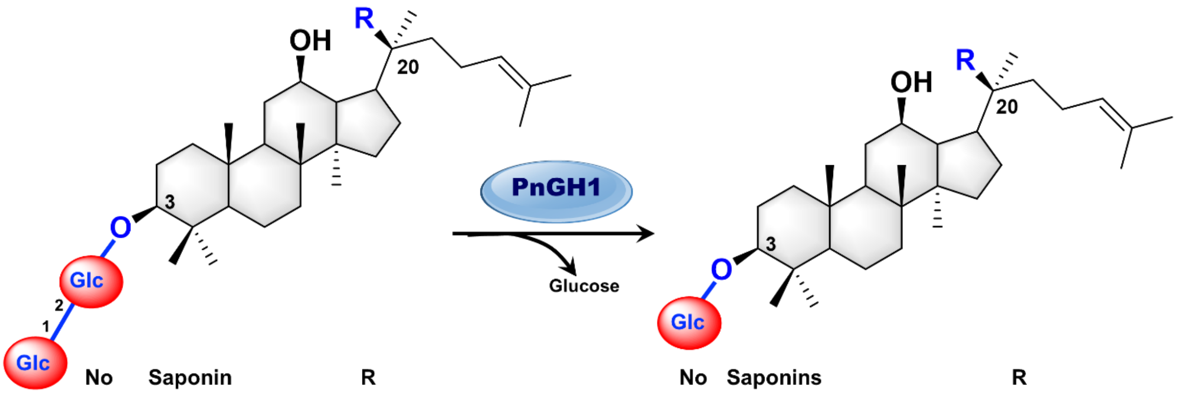


| No | Saponin | R | No | Saponins | R |
|---|---|---|---|---|---|
| 1 | Rb1 | OGlc(6-1)Glc | 1a | XVII | OGlc(6-1)Glc |
| 2 | Rc | OGlc(6-1)Araf | 2a | Fe | OGlc(6-1)Araf |
| 3 | Rb2 | OGlc(6-1)Arap | 3a | Rd2 | OGlc(6-1)Arap |
| 4 | Rb3 | OGlc(6-1)Xyl | 4a | Fd | OGlc(6-1)Xyl |
| 6 | Ra2 | OGlc(6-1)Araf(2-1)Xyl | 6a | PN02 | OGlc(6-1)Araf(2-1)Xyl |
| 7 | Ra3 | OGlc(6-1)Glc(3-1)Xyl | 7a | PN03 | OGlc(6-1)Glc(3-1)Xyl |
| 8 | Ra1 | OGlc(6-1)Arap(4-1)Xyl | 8a | PN01 | OGlc(6-1)Arap(4-1)Xyl |
| 9 | Rd | OGlc | 9a | F2 | OGlc |
| 10 | Rg3 | OH | 10a | Rh2 | OH |

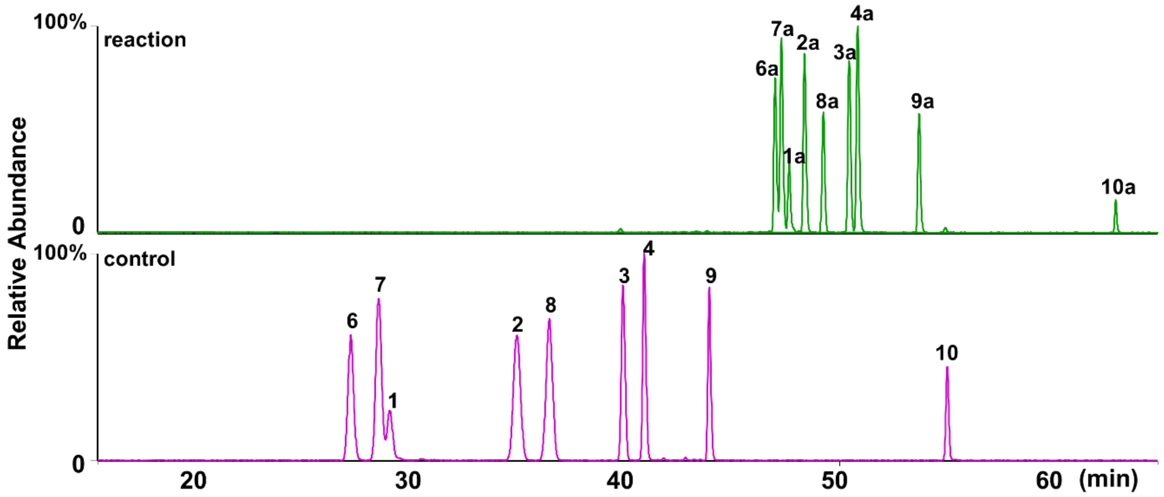


**Supplementary Fig. 12** Schemes and MS spectra of *β* (1→2) glucosidic linkage hydrolysis reactions of PnGH1 using 20(*S*)-PPD type ginsenosides **1**–**4** and **6**–**10** as substrates yielding the deglucosylated producta **1a**–**4a** and **6a**–**10a**, respectively.

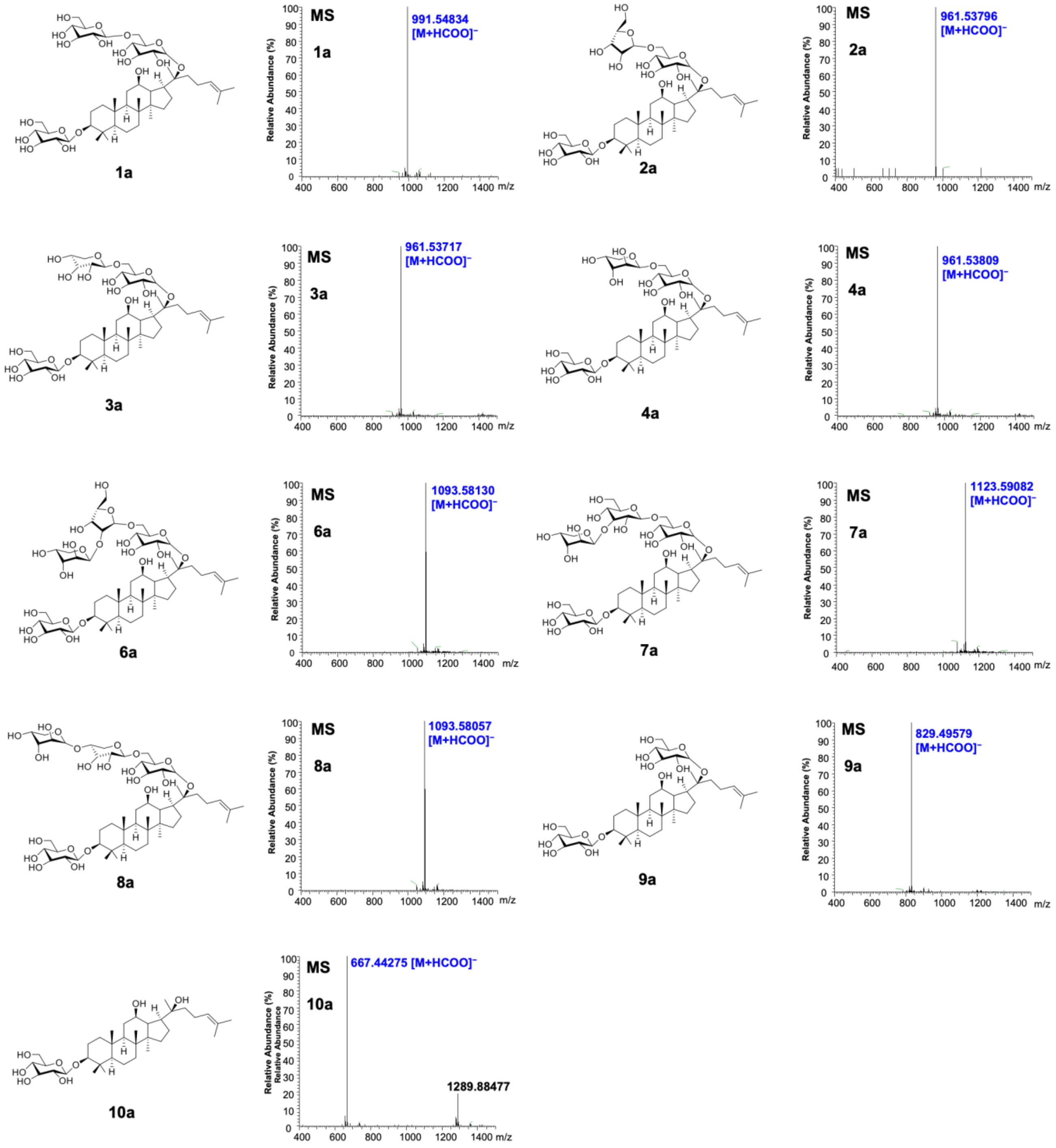


**Supplementary Fig. 13** Chemical structures and MS spectra of the hydrolyzed products **1a**–**4a** and **6a**–**10a**.

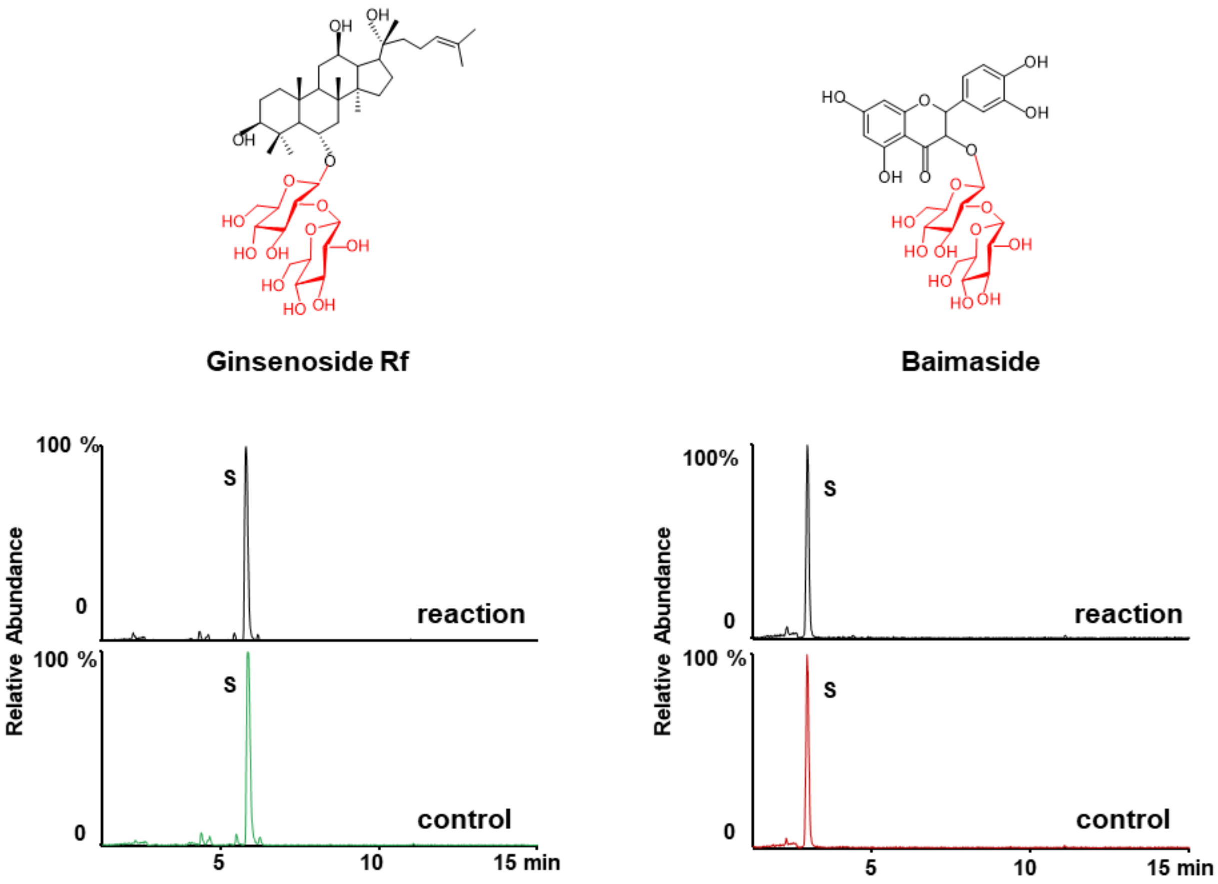


**Supplementary Fig. 14** LC-MS analysis of hydrolysis reactions catalyzed by PnGH1 using ginsenoside Rf (left), and baimaside (right) as the substrate (S), respectively.

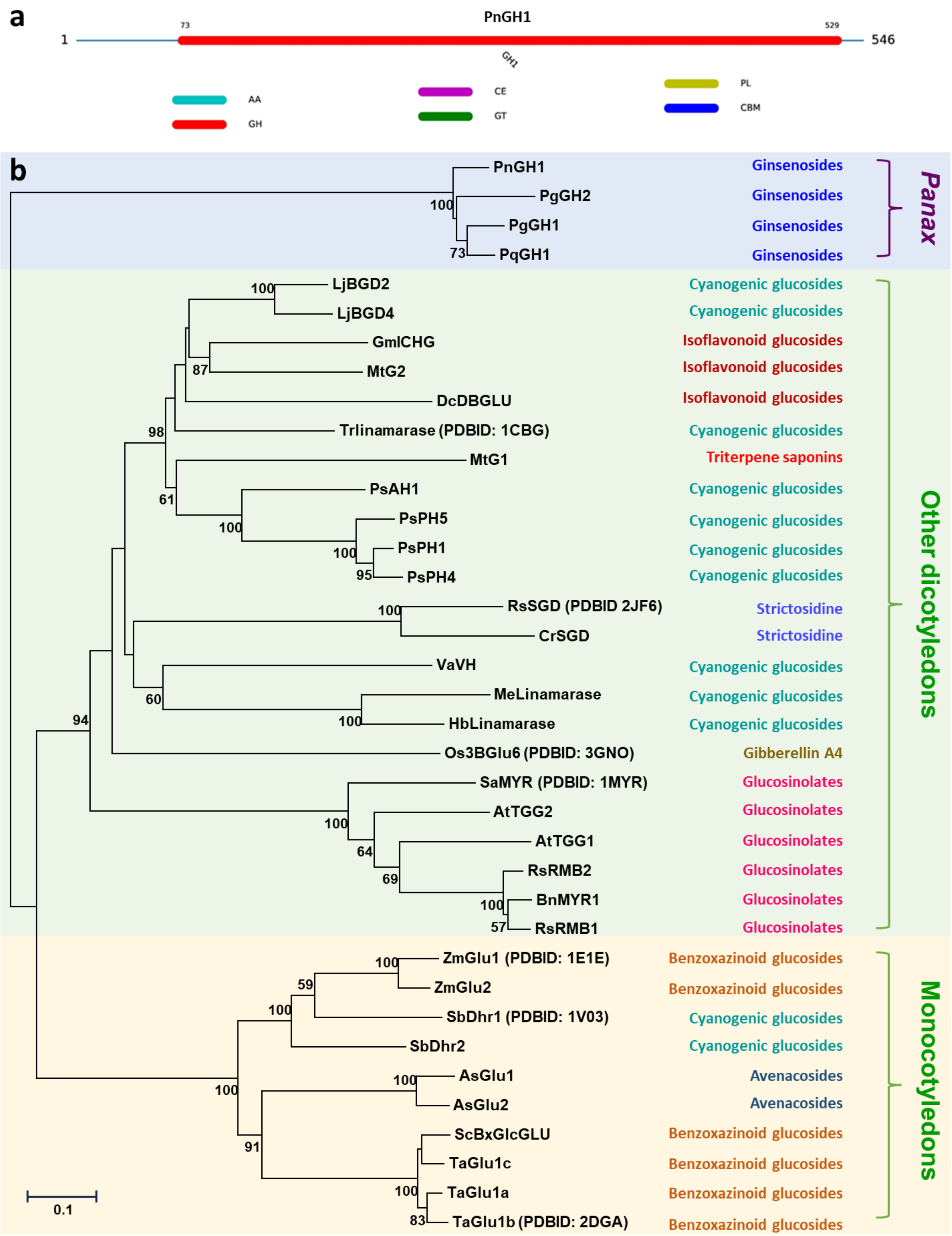


**Supplementary Fig. 15** (**a**) Carbohydrate Active Enzyme (CAZy) database annotation of PnGH1. (**b**) Phylogenetic analysis of *β*-glucosidases involved in the bioactivation of defense compounds. The

phylogenetic tree was constructed using the Neighbor-Joining method based on the Jones, Taylor, and Thorton (JTT) model[1] by MEGA7 software[2]. The tree was bootstrapped with 1000 iterations. The bootstrap values are designated by percentages. Node values lower than 50% were cut off. Other *β*-glucosidases involve ed in the bioactivation of defense compounds which were used for phylogenetic analyses were selected based on previously reported literature[3-5], including **LjBGD2** and **LjBGD4** (EU10844, EU10845): *Lotus japonicus* *β*-glucosidases[4]; **GmICHG** (AB259819): *Glycine max* isoflavone conjugate-hydrolyzing *β*-glucosidase[6]; **MtG1** (EU078901) and **MtG2** (EU078904): *Medicago truncatula* *β*-glucosidases[7]; **DcBDGLU** (AF163097): *Dalbergia cochinchinensis* Pierre dalcochinase[8]; **Trlinamarase** (PDBID: 1CBG): *Trifolium repens* linamarase[9]; **PsAH1** (X56733), **PsPH1** (AF414608), **PsPH4** (AF411928) and **PsPH5** (AF413213): *Prunus serotina* amygdalin hydrolase and prunasin hydrolase isozymes[10]; **RsSGD** (CAC83098): *Rauvolfia serpentina* strictosidine *β*-glucosidases orthologs[11]; **CrSGD** (AAF28800): *Catharanthus roseus* strictosidine *β*-glucosidases orthologs[12]; **VaVH** (DQ371927): *Vicia angustifolia* vicianin hydrolase[13]; **MeLinamarase** (AAB22162.1): *Manihot esculenta* Crantz linamarase[14,15]; **HbLinamarase** (AAO49267.1): *Hevea brasiliensis* linamarase[16]; **Os3BGlu6** (PDBID: 3GNO): *Oryza sativa* gibberellin *β*-glucosidase[17]; **SaMYR** (PDBID: 1MYR): *Sinapis alba* myrosinase[18]; **AtTGG1** (NP_851077.1) and **AtTGG2** (NP_568479.1): *Arabidopsis thaliana* myrosinases[19]; **BnMYR1** (NP_001302796.1): *Brassica napus* myrosinase[20]; **RsRMB1** (AB042186) and **RsRMB2** (AB042186): Radish *Raphanus sativus* myrosinases[21]; **ZmGlu1** (U25157) and **ZmGlu2** (Q41761.1): *Zea mays* *β*-glucosidases[22]; **SbDhr1** (PDBID: 1V03) and **SbDhr2** (AAK49119.1): *Sorghum bicolor* Moench dhurrinases[23,24]; **AsGlu1** (Q38786.1) and **AsGlu2** (Q9ZP27.1): *Avena sativa* avenacosidases[25,26]; **ScBxGlcGLU** (AF293849): *Secale cereale* *β*-glucosidase[27]; **TaGlu1a** (AB100035), **TaGlu1b** (AB236422) and **TaGlu1c** (AB236423): *Triticum aestivum* L. *β*-glucosidases[28]. The accession numbers or PDBID of these genes were noted in the bracket.

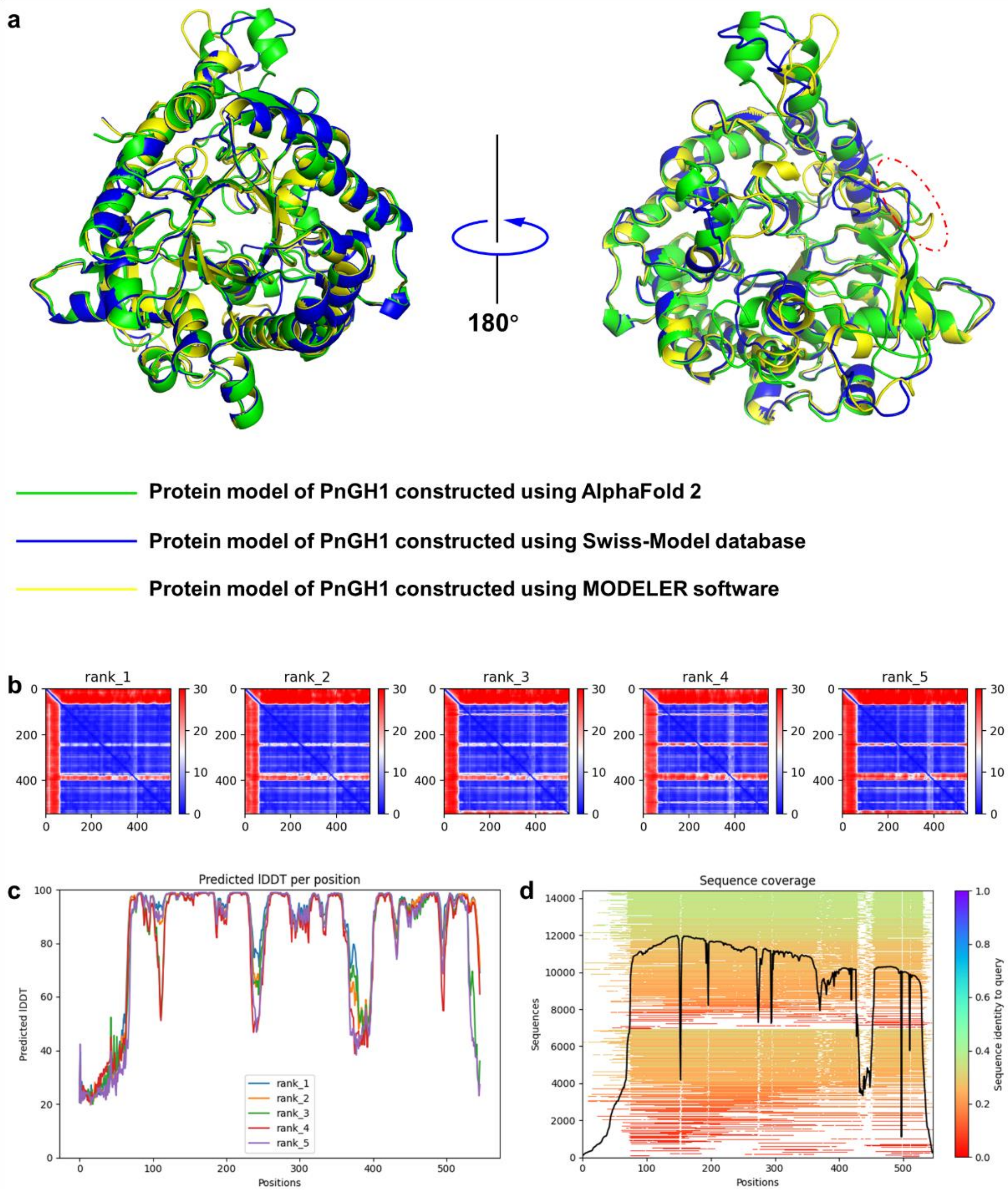


**Supplementary Fig. 16** (**a**) Overall structures of protein models of PnGH1 constructed using different methods. Regions showing low accuracy in AlphaFold2 calculated model was labeled in red circle. Structure of sequences 1–70 at N-terminal were not shown. (**b–d**) Evaluation of PnGH1 model constructed by AlphaFold2 using predicted aligned error (**b**), pLDDT (**c**) and sequence coverage parameters (**d**). Note: The overall structure of the obtained three models showed high similarity except for some random coil regions. Whereas the AlphaFold2 constructed model exhibited low prediction accuracy around residues 360-400 of which some residues are potentially involved in the hydrolysis process. Thus, the homology modeling structure constructed by MODELER software was used for structural analysis.

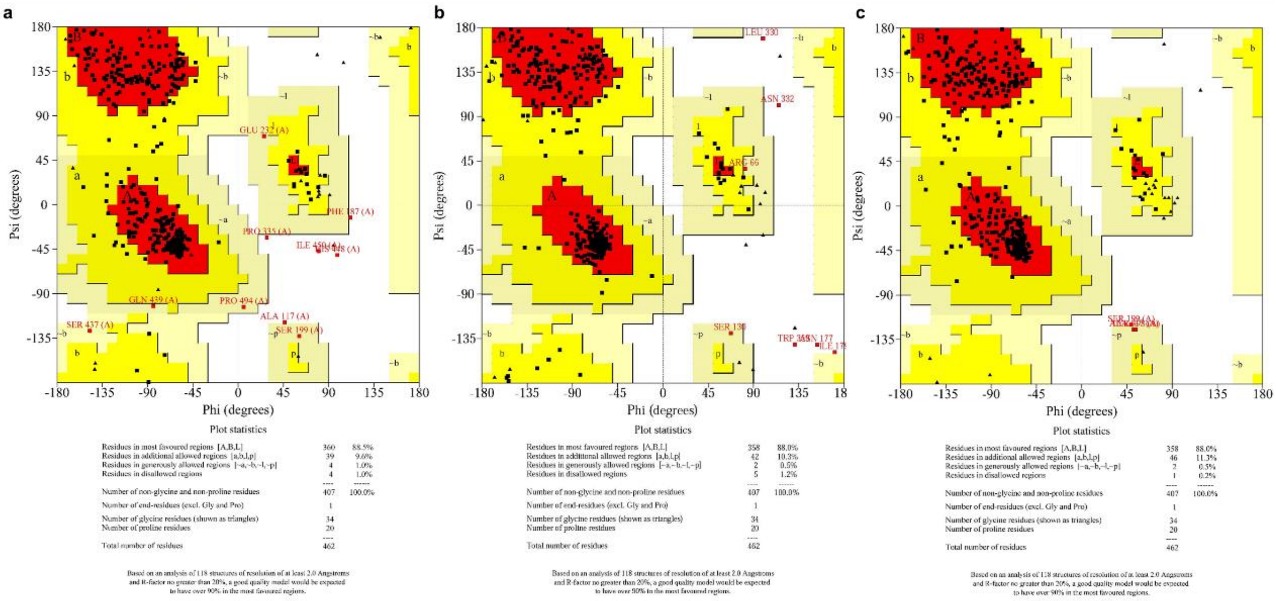


**Supplementary Fig. 17** PROCHECK Ramachandran plot of PnGH1 model (70–531aa) constructed using Swiss-Model database (**a**), MODELER software v.9.14 (**b**) and AlphaFold 2 (**c**).

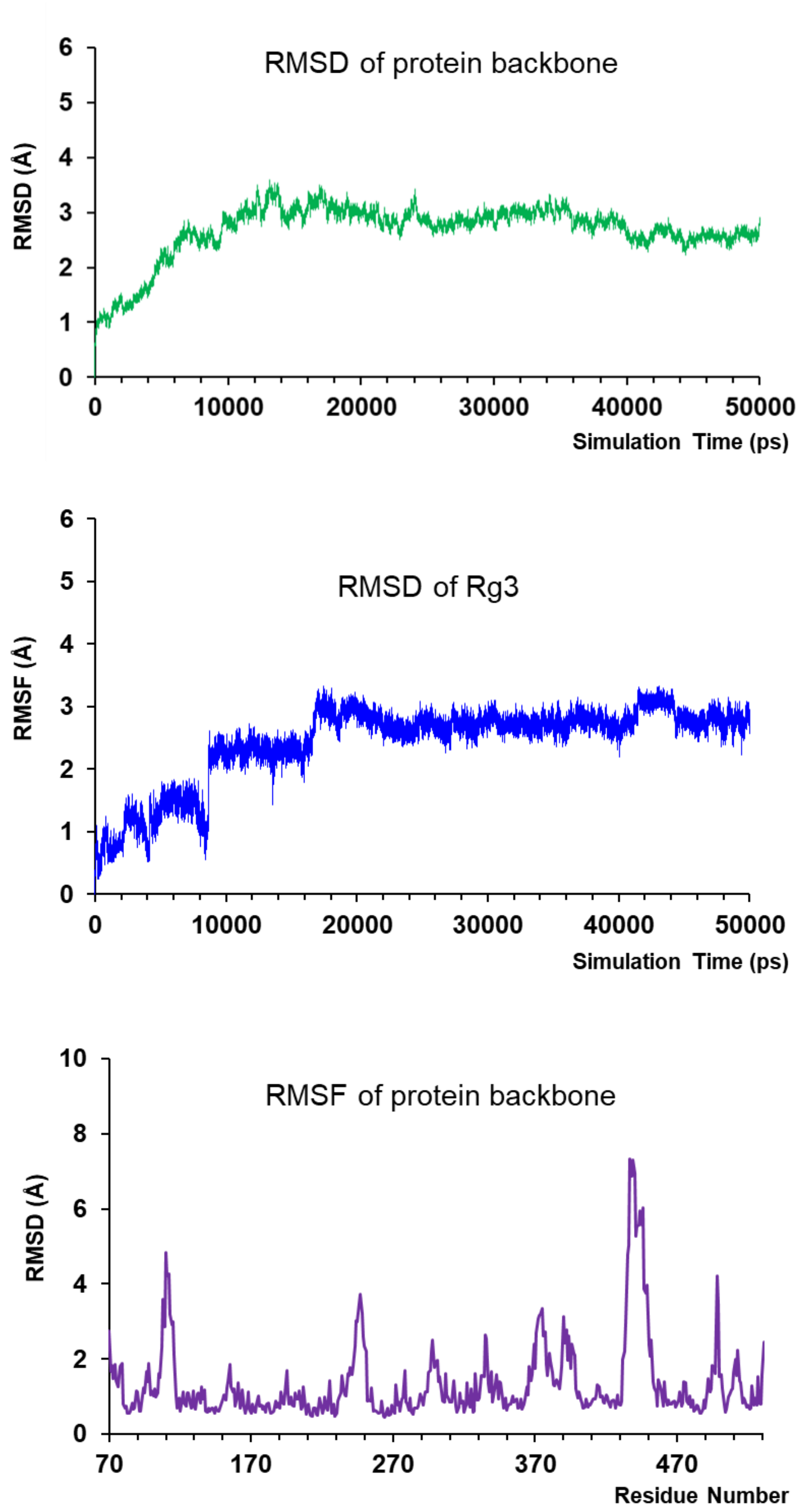


**Supplementary Fig. 18** RMSD, RMSF of PnGH1 protein structure model, and RMSD of substrate Rg3 during the 50 ns MD simulations of PnGH1–Rg3 complex.

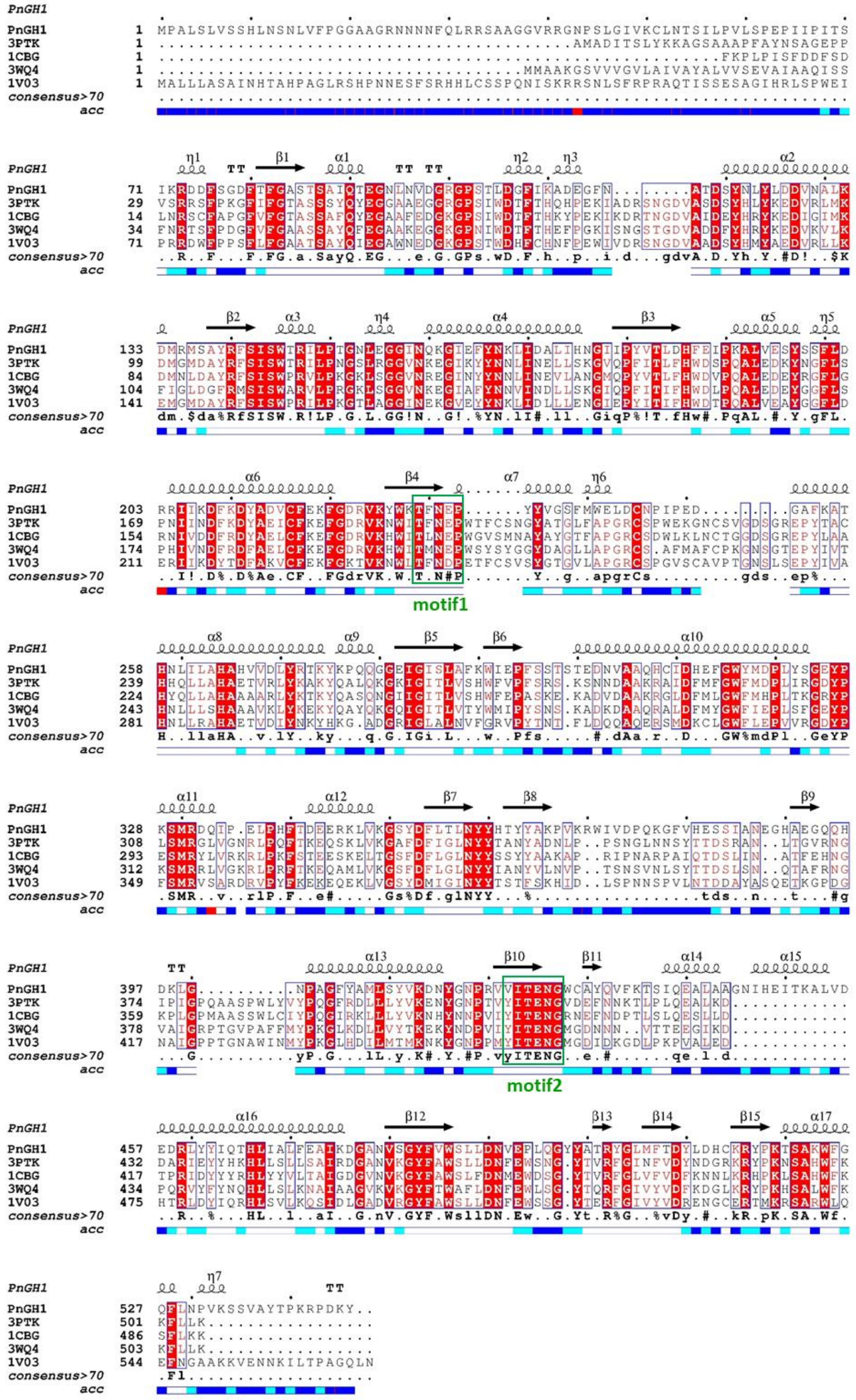


**Supplementary Fig. 19** Amino acid alignment of PnGH1 and the related *β*-glycosidases. TFN(E/D)P motif (motif1) and (V/Y) ITENG motif (motif2) were marked in green frames.

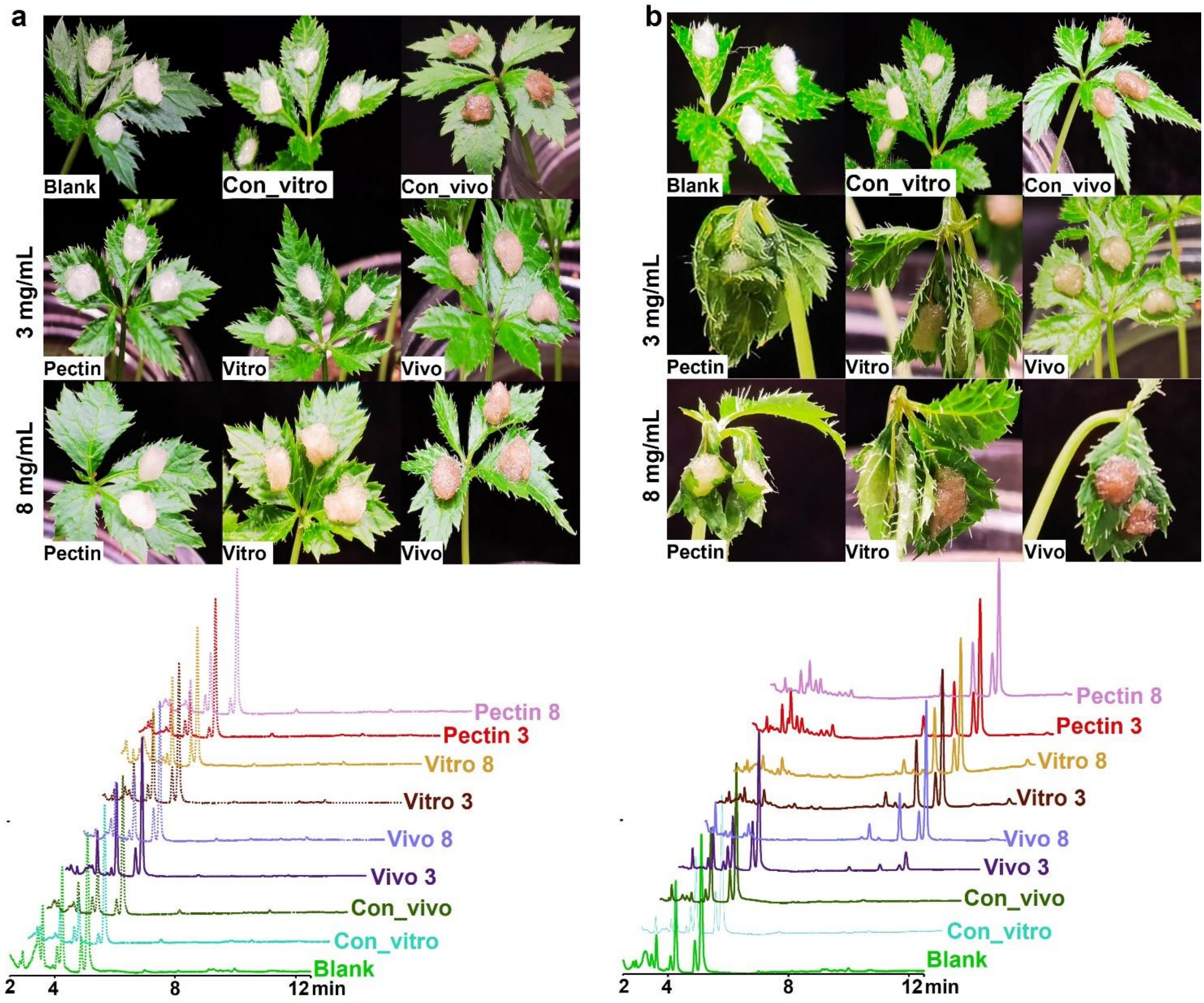


**Supplementary Fig. 20** Representative photographs (up) and the HPLC profile (down) of PNL inoculated with controls and induced-exoenzymes at 0 (**a**) and 5 DPI (**b**). The sterile absorbent cotton containing the exoenzymes induced by PNL *in vivo* and *in vitro*, and pectin as well (3 and 8 mg $mL^{-1}$) was placed on a 4-week-old *P. notoginseng* leaves that were pre-wounded by a fine needle. Leaves treated with sterile water was used as blank control, the crude exoenzyme extracted from the steamed PNL without the fungal inoculation and *M. acerina*-inoculated medium without pectin or PNL tissue were used as *in vivo* control (Con_vivo, 8 mg $mL^{-1}$) and *in vitro* control (Con_vitro, 8 mg $mL^{-1}$), respectively.

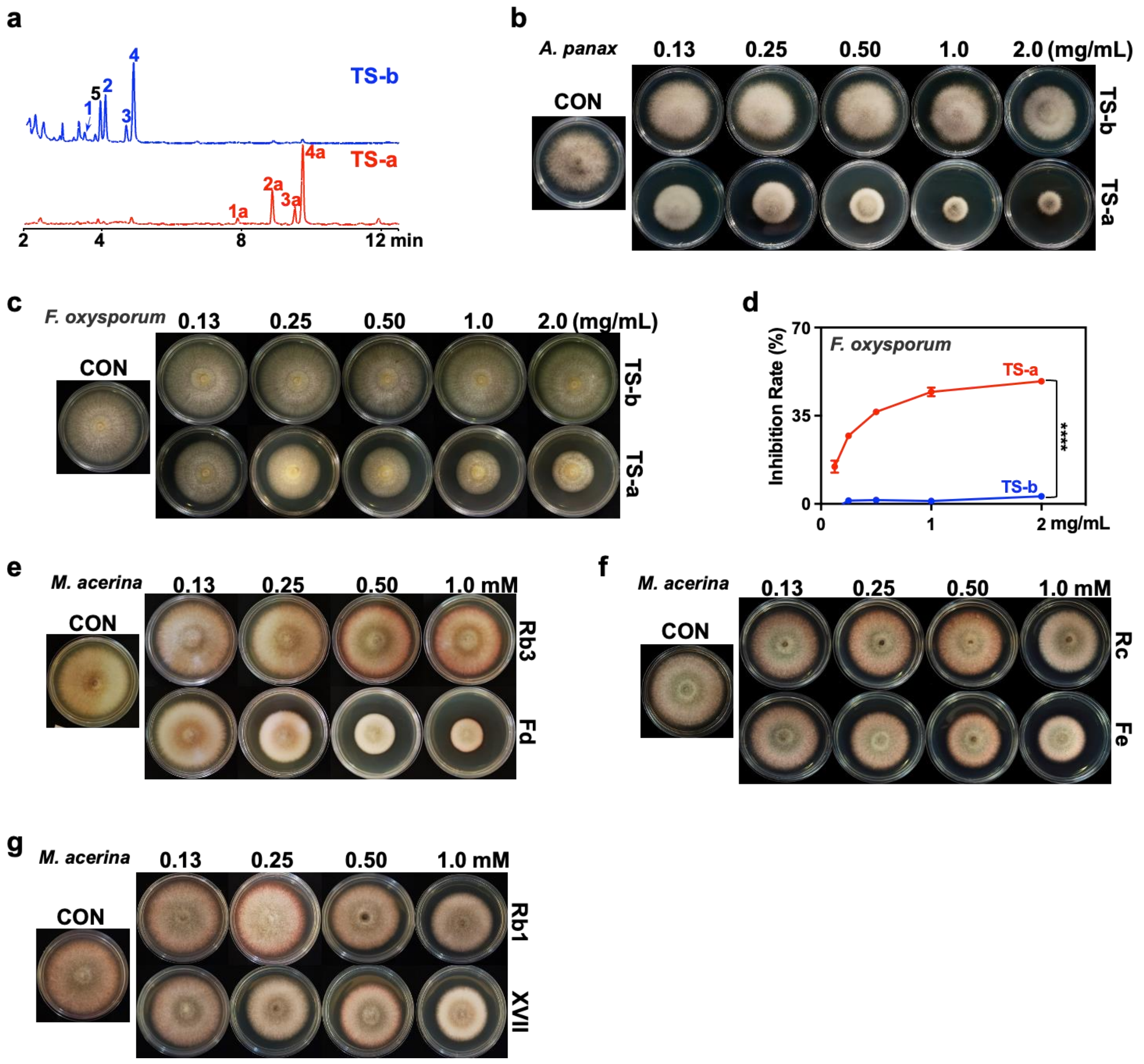


**Supplementary Fig. 21 (a)** HPLC-UV profiles of the refined total saponin extracts of PNL before (TS-b) and after (TS-a) hydrolysis. **1**, ginsenoside Rb1; **2**, ginsenoside Rc; **3**, ginsenoside Rb2; **4**, ginsenoside Rb3; **5**, notoginsenoside Fc; **1a**, gypenoside XVII; **2a**, notoginsenoside Fe; **3a**, ginsenosides Rd2; **4a**, notoginsenosides Fd. **b-d**, *In vitro* antifungal activity of TS-b and TS-b against (**b**) *A. panax* and (**c**) *F. oxysporu*m and (**d**) corresponding growth inhibition ratio. **e-g**, *In vitro* antifungal activity of (**e**) Rb3/Fd, (**f**) Rc/Fd and (**g**) Rb1/XVII against *M. acerina.* **d** n=4 biologically independent samples. The data are expressed as mean ± SD. Statistical significance was calculated using Student's *t*-test. ****, $p < 0.0001$.

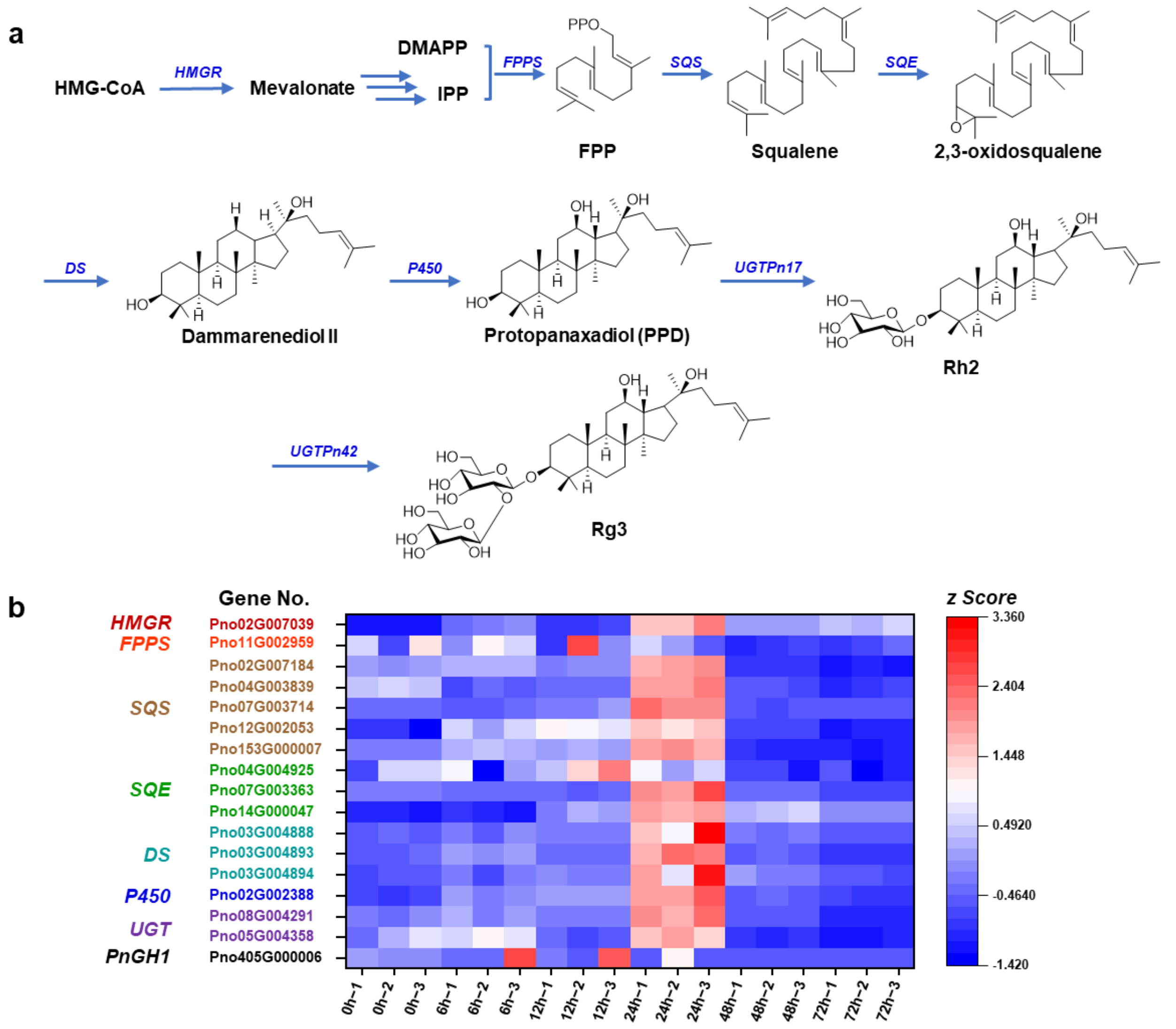


**Supplementary Fig. 22** (**a**) Biosynthesis of PPD-type ginsenosides in *P. notoginseng* (ginsenoside Rg3 as an example). Abbreviations: 3-hydroxy-3-methylglutaryl coenzyme A (*HMG-CoA*); HMG-CoA reductase (*HMGR*), dimethylallyl diphosphate (*DMAPP*), isopentenyl diphosphate (*IPP*), farnesyl diphosphate (*FPP*), farnesyl diphosphate synthase (*FPPS*), squalene synthase (*SQS*), squalene epoxidase (*SQE*), dammarenediol-II synthase (*DS*), cytochrome P450 (*P450*), UDP-glycosyltransferase (*UGT*) (**b**) Expression levels of *PnGH1* and genes involved in ginsenosides biosynthesis after being infected by the necrotrophic fungus *M. acerina* at different time points. Ginsenosides biosynthesis-related genes were searched through homology blast based on previously reported sequences[29-33]. The expression values of each gene were normalized as z-scores using FPKM values and visualized as a heatmap in Origin 2019 software.

**Supplementary Table 1.** Information of four candidate glycosidase genes from *P. notoginseng*.

| **Gene locus number** | **TRINITY_DN19051_c0_g1** | **TRINITY_DN22617_c3_g1** | **TRINITY_DN5550_c0_g1** |
|---|---|---|---|
| Matched peptides | 15 | 4 | 7 |
| Matched queries | 227 | 51 | 121 |
| coverage | 39.93% | 21.16% | 35.23% |
| SEQUEST HT score | **509.45** | **14.85** | **31.66** |
| Automatic annotation | Glycosyl hydrolase family 1 | Glycosyl hydrolase family 18 | Glycosyl hydrolase family 17 |
| Gene name | **PnGH1** | **PnGH2** | **PnGH3** |

**Supplementary Table 2.** Detailed interactions between substrate **10** and key residues in PnGH1.

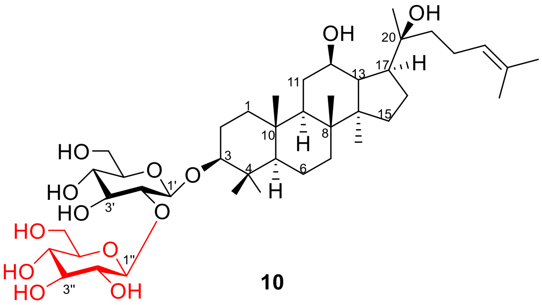

**10**

| Hydrophobic Interactions | | Distance |
|---|---|---|
| **Substrate 10** | **Residues** | |
| C6 | CE2 atom of Y234 | 3.4 Å |
| C29 | CD2 atom of Y234 | 3.9 Å |
| **Hydrogen-bond interactions** | | **Distances** |
| **Substrate 10** | **Residues** | |
| 4″-OH | OE1 atom of Q90 | 2.9 Å |
| 6″-OH | NE2 atom of Q90 | 3.5 Å |
| 4″-OH | NE2 atom of H186 | 2.8 Å |
| 2″-OH | ND2 atom of N231 | 2.8 Å |
| 3″-OH | ND2 atom of N231 | 3.5 Å |
| 12-OH | NZ atom of K398 | 2.9 Å |
| 2″-OH | OE1 atom of E425 | 3.3 Å |
| 2″-OH | OE2 atom of E425 | 3.0 Å |
| 6′-OH | OE1 atom of E493 | 2.7 Å |
| 6″-OH | OE2 atom of E493 | 2.6 Å |
| 6″-OH | OH atom of Y503 | 2.9 Å |

**Supplementary Table 3.** Analysis methods used in this study.

| | |
|---|---|
| **Method 1** | Agilent 1290 Infinity II UPLC system.<br>A Kinetex $C_{18}$ column (100 mm × 4.6 mm, 2.6 μm, Phenomenex)<br>The mobile phases consisted of water (A) and acetonitrile (B) at a flow rate of 0.6 mL $min^{-1}$. The linear gradient program was adopted as follows: 0-5 min, 34 % B; 5-6 min, 36-38.5%B; 6-12 min, 38.5-45% B; 12-12.5 min, 34 %B. The detection wavelength and column temperature were set at 203 nm and 40°C, respectively. |
| **Method 2** | Agilent 1260 Series HPLC system.<br>Agilent $C_{18}$ Column (2.1 mm I.D. × 100 mm, 1.8 μm).<br>The mobile phase consisted of solvent A (0.5 ‰ trifluoroacetic acid) and solvent B (acetonitrile) at 1.0 mL $min^{-1}$ with a gradient elution procedure as follows: 0-3 min, 15% B, 3-12min, 15-35% B, 12-18 min, 35-40% B, 18-23 min, 40-50% B, 23-25 min, 50-95% B, 25-35 min, 95-15% B. |
| **Method 3** | Nano-LC-LTQ Orbitrap Velos Pro MS (ThermoFisher Scientific).<br>The trap column was packed with 5 μm $C_{18}$ reversed phase material.<br>The analytical column (75 μm × 10 cm) was packed with 3 μm $C_{18}$ reversed phase material.<br>EASY-nLC II system was used to generate the HPLC gradient 2–40% B in 0−70 min, 40–95% B in 70−75 min, and 95% B in 75−95 min (A: 0.1% formic acid in water; B: 0.1% formic acid in acetonitrile).<br>The eluted peptides were sprayed into a Nano-LC-LTQ Orbitrap Velos Pro mass spectrometer equipped with a nano-ESI ion source. Ion scanning was performed in the Orbitrap analyzer in the scan range of m/z 300–1,500 and at a resolution of 60,000 (FWHM). CID collision energy was set to 35% for the generation of $MS^2$ spectra. |
| **Method 4** | DIONEX Ultimate 3000 UHPLC system coupled with an LTQ-Orbitrap XL mass spectrometer (Thermo Fisher Scientific, MA, USA).<br>The chromatographic separation was achieved on a Waters ACQUITY UPLC BEH C8 column (150 mm × 2.1 mm, 1.7 μm, Waters, MA, USA) with a solvent system of 0.1% aqueous formic acid solution (*v/v*, A) and ACN (B) at a flow rate of 0.4 mL $min^{-1}$ using a gradient as follows: 0-25 min, 15-28% B, 25-50 min, 28-50% B, 50-65 min, 50-95% B.<br>The MS parameters were optimized in negative ionization mode as follows: ion spray voltage of -4.0 kV, capillary temperature of 350 °C, capillary voltage of -30V, tube lens voltage of -110 V, sheath gas flowrate of 65 arbitrary units, auxiliary gas flow |

| | |
|---|---|
| | rate of 20 arbitrary units. HRMS and data-dependent scan (DDS) MS/MS data were acquired from *m*/*z* 400 to 1800.<br>All data were analyzed by Xcalibur 2.1 software (Thermo Fisher Scientific, MA, USA). |

**Supplementary Table 4.** Primers used in this study.

| No. | Amplicons | Sequences (5′−3′) |
|---|---|---|
| 1 | *PnGH1*-F | ATGCCTGCTCTCAGCCTTGTT |
| 2 | *PnGH1*-R | TTAAGATGAACTAATATTTGTCGGGG |
| 3 | *PnGH2*-F | ATGGCAACCCATTCACATCT |
| 4 | *PnGH2*-R | TCAGATACTACCCTTTATAGCAGAACTATA |
| 5 | *PnGH3*-F | ATGGCTACATTTCATTCCGTC |
| 6 | *PnGH3*-R | TCAGTTGAAACTAAGTTGGTACTTTGA |
| 7 | *PgGH1*-F | ATGGCTGCTCTCAACCTAATTGTTTCTTCCCAT |
| 8 | *PgGH1*-R | CTAATATTCGTGGGGGCGTTTAGGTGTGTAT |
| 9 | *PgGH2*-F | ATGGCTGCTCTCAAACTTGTTTC |
| 10 | *PgGH2*-R | GTTTAAATAGTCCAAGTAATCTGTAAACATCA |
| 11 | *PqGH1*-F | ATGGCTCTCAACCTAATTG |
| 12 | *PqGH1*-R | CTAATATTTATCGGGGCGTTTAGG |
| 13 | *PnGH1-Sac* I-*F* | GAGAACACGGGGGACGAGCTCATGCCTGCTCTCAGCCTTGTT |
| 14 | *PnGH1-Xba* I-*R* | GCTCACCATGTCGACTCTAGAATATTTGTCGGGGCGTTTAGG |
| 15 | Q90A-F | GGTGCCTCCACTTCTGCTATC**GC**GACCGAAGGAAATCT |
| 16 | Q90A-R | **GC**CGATAGCAGAAGTGGAGGCACCAAACGTGAAAT |
| 17 | H186A-F | CTTATGTGACCTTAGAC**GC**CTTCGAAATACCGAAAGCTCT |
| 18 | H186A-R | **GC**GTCTAAGGTCACATAAGGAATTATTCCATT |
| 19 | N231A-F | CGTGTGAAATACTGGAAGACATTT**GC**CGAGCCATATTATGTGG |
| 20 | N231A-R | **GC**AAATGTCTTCCAGTATTTCACACGGTCGCCAAATT |
| 21 | E232A-F | TGGAAGACATTTAACG**C**GCCATATTATGTGGGT |
| 22 | E232A-R | **G**CGTTAAATGTCTTCCAGTATTTCACACGGTC |
| 23 | E232D-F | TGGAAGACATTTAACGA**C**CCATATTATGTGGGT |
| 24 | E232D-R | **G**TCGTTAAATGTCTTCCAGTATTTCACACGGTC |
| 25 | Y234A-F | CATTTAACGAGCCATAT**GC**TGTGGGTTCATTTATG |
| 26 | Y234A-R | **GC**ATATGGCTCGTTAAATGTCTTCCAG |
| 27 | K398A-F | CGCAGAAGGACAACAGCATGAT**GC**ATTGGGGAATCCTGCAGG |
| 28 | K398A-R | **GC**ATCATGCTGTTGTCCTTCTGCGTGGCCTTCATT |
| 29 | E425A-F | CTAGAGTTGTAATTACTG**C**AAATGGATGGTGT |
| 30 | E425A-R | **G**CAGTAATTACAACTCTAGGGTTTCCATAATT |
| 31 | E425D-F | CTAGAGTTGTAATTACTGA**C**AATGGATGGTGT |
| 32 | E425D-R | **G**TCAGTAATTACAACTCTAGGGTTTCCATAATT |
| 33 | E493A-F | TGGATAACGTGG**C**ACCGCTTCAGGGGTATT |
| 34 | E493A-R | **G**CCACGTTATCCAACAGCGACCACACAAAATAT |
| 35 | E493D-F | TGGATAACGTGGA**C**CCGCTTCAGGGGTATT |
| 36 | E493D-R | **G**TCCACGTTATCCAACAGCGACCACACAAAATAT |
| 37 | Y503A-F | TTATGCCACACGA**GC**TGGGTTGATGTTCACAG |
| 38 | Y503A-R | **GC**TCGTGTGGCATAATACCCCTGAAG |

Note: mutagenesis sites are highlighted in bold format.

## References


1. Jones, D. T., Taylor, W. R. & Thornton, J. M. The rapid generation of mutation data matrices from protein sequences. *Comput. Appl. Biosci.* **8**, 275–282 (1992).

2. Kumar, S., Stecher, G. & Tamura, K. MEGA7: Molecular Evolutionary Genetics Analysis Version 7.0 for Bigger Datasets. *Mol. Biol. Evol.* **33**, 1870–1874 (2016).

3. Lacchini, E. *et al.* The saponin bomb: a nucleolar-localized β-glucosidase hydrolyzes triterpene saponins in *Medicago truncatula*. *New Phytol.* **239**, 705–719 (2023).

4. Morant, A. V. *et al.* The beta-glucosidases responsible for bioactivation of hydroxynitrile glucosides in *Lotus japonicus*. *Plant Physiol.* **147**, 1072–1091 (2008).

5. Morant, A. V. *et al.* beta-Glucosidases as detonators of plant chemical defense. *Phytochemistry* **69**, 1795–1813 (2008).

6. Suzuki, H. *et al.* An isoflavone conjugate-hydrolyzing beta-glucosidase from the roots of soybean (*Glycine max*) seedlings: purification, gene cloning, phylogenetics, and cellular localization. *J. Biol. Chem.* **281**, 30251–30259 (2006).

7. Naoumkina, M. *et al.* Different mechanisms for phytoalexin induction by pathogen and wound signals in *Medicago truncatula*. *Proc. Natl. Acad. Sci. U S A* **104**, 17909–17915 (2007).

8. Ketudat Cairns, J. R. *et al.* Sequence and expression of Thai Rosewood beta-glucosidase/beta-fucosidase, a family 1 glycosyl hydrolase glycoprotein. *J. Biochem.* **128**, 999–1008 (2000).

9. Barrett, T., Suresh, C. G., Tolley, S. P., Dodson, E. J. & Hughes, M. A. The crystal structure of a cyanogenic beta-glucosidase from white clover, a family 1 glycosyl hydrolase. *Structure* **3**, 951–960 (1995).

10. Zhou, J., Hartmann, S., Shepherd, B. K. & Poulton, J. E. Investigation of the microheterogeneity and aglycone specificity-conferring residues of black cherry prunasin hydrolases. *Plant Physiol.* **129**, 1252–1264 (2002).

11. Guirimand, G. *et al.* Strictosidine activation in Apocynaceae: towards a "nuclear time bomb"? *BMC Plant Biol.* **10**, 182 (2010).

12. Geerlings, A., Ibañez, M. M., Memelink, J., van Der Heijden, R. & Verpoorte, R. Molecular cloning and analysis of strictosidine beta-D-glucosidase, an enzyme in terpenoid indole alkaloid biosynthesis in *Catharanthus roseus*. *J. Biol. Chem.* **275**, 3051–3056 (2000).

13. Ahn, Y. O., Saino, H., Mizutani, M., Shimizu, B. & Sakata, K. Vicianin hydrolase is a novel cyanogenic beta-glycosidase specific to beta-vicianoside (6-O-alpha-L-arabinopyranosyl-beta-D-glucopyranoside) in seeds of *Vicia angustifolia*. *Plant Cell Physiol.* **48**, 938–947 (2007).

14. Hughes, M. A. *et al.* A molecular and biochemical analysis of the structure of the cyanogenic beta-glucosidase (linamarase) from cassava (*Manihot esculenta* Cranz). *Arch. Biochem. Biophys.* **295**, 273–279 (1992).

15. Keresztessy, Z., Brown, K., Dunn, M. A. & Hughes, M. A. Identification of essential active-site

residues in the cyanogenic beta-glucosidase (linamarase) from cassava (*Manihot esculenta* Crantz) by site-directed mutagenesis. *Biochem. J.* **353**, 199–205 (2001).

16. Selmar, D., Lieberei, R., Biehl, B. l. & Voigt, J. r. Hevea linamarase—a nonspecific β-glycosidase. *Plant Physiol.* **83**, 557–563 (1987).

17. Hua, Y., Sansenya, S., Saetang, C., Wakuta, S. & Ketudat Cairns, J. R. Enzymatic and structural characterization of hydrolysis of gibberellin A4 glucosyl ester by a rice *β*-D-glucosidase. *Arch. Biochem. Biophys.* **537**, 39–48 (2013).

18. Burmeister, W. P. *et al.* The crystal structures of *Sinapis alba* myrosinase and a covalent glycosyl-enzyme intermediate provide insights into the substrate recognition and active-site machinery of an S-glycosidase. *Structure* **5**, 663–675 (1997).

19. Barth, C. & Jander, G. Arabidopsis myrosinases TGG1 and TGG2 have redundant function in glucosinolate breakdown and insect defense. *Plant J.* **46**, 549–562 (2006).

20. Chen, S. & Halkier, B. A. Functional expression and characterization of the myrosinase MYR1 from *Brassica napus* in *Saccharomyces cerevisiae*. *Protein Expr. Purif.* **17**, 414–420 (1999).

21. Hara, M., Fujii, Y., Sasada, Y. & Kuboi, T. CDNA cloning of radish (*Raphanus sativus*) myrosinase and tissue-specific expression in root. *Plant Cell Physiol.* **41**, 1102–1109 (2000).

22. Czjzek, M. *et al.* Crystal structure of a monocotyledon (maize ZMGlu1) beta-glucosidase and a model of its complex with p-nitrophenyl beta-D-thioglucoside. *Biochem. J.* **354**, 37–46 (2001).

23. Hösel, W., Tober, I., Eklund, S. H. & Conn, E. E. Characterization of beta-glucosidases with high specificity for the cyanogenic glucoside dhurrin in *Sorghum bicolor* (L.) moench seedlings. *Arch. Biochem. Biophys.* **252**, 152–162 (1987).

24. Verdoucq, L. *et al.* Structural determinants of substrate specificity in family 1 beta-glucosidases: novel insights from the crystal structure of sorghum dhurrinase-1, a plant beta-glucosidase with strict specificity, in complex with its natural substrate. *J. Biol. Chem.* **279**, 31796–31803 (2004).

25. Gus-Mayer, S., Brunner, H., Schneider-Poetsch, H. A. & Rüdiger, W. Avenacosidase from oat: purification, sequence analysis and biochemical characterization of a new member of the BGA family of beta-glucosidases. *Plant Mol. Biol.* **26**, 909–921 (1994).

26. Kim, Y. W., Kang, K. S., Kim, S. Y. & Kim, I. S. Formation of fibrillar multimers of oat beta-glucosidase isoenzymes is mediated by the As-Glu1 monomer. *J. Mol. Biol.* **303**, 831–842 (2000).

27. Nikus, J., Esen, A. & Jonsson, L. M. Cloning of a plastidic rye (*Secale cereale*) *β*-glucosidase cDNA and its expression in *Escherichia coli*. *Physiol. Plantarum* **118**, 337–345 (2003).

28. Sue, M. *et al.* Molecular and structural characterization of hexameric beta-D-glucosidases in wheat and rye. *Plant Physiol.* **141**, 1237–1247 (2006).

29. Luo, H., *et al.* Analysis of the transcriptome of *Panax notoginseng* root uncovers putative triterpene saponin-biosynthetic genes and genetic markers. *BMC Genomics* **12 Suppl 5**, S5 (2011).

30. Niu, Y., *et al.* Expression profiling of the triterpene saponin biosynthesis genes FPS, SS, SE, and DS in the medicinal plant *Panax notoginseng*. *Gene* **533**, 295-303 (2014).

31. Xu, S., *et al.* Longitudinal expression patterns of HMGR, FPS, SS, SE and DS and their correlations with saponin contents in green-purple transitional aerial stems of *Panax notoginseng*. *Ind. Crop Prod.* **119**, 132-143 (2018).

32. Jiang, Z., *et al.* Key glycosyltransferase genes of *Panax notoginseng*: identification and engineering yeast construction of rare ginsenosides. *ACS Synth. Biol.* **11**, 2394-2404 (2022).

33. Li, Y., *et al.* Characterization of a group of UDP-glycosyltransferases involved in the biosynthesis of triterpenoid saponins of *Panax notoginseng. ACS Synth. Biol.* **11**, 770-779 (2022).
