# Supplementary material for: Discovery of plant chemical defence mediated by a two-component system involving β-glucosidase in Panax species
Source: Nat Commun. 2024 Jan 18;15:602. doi: 10.1038/s41467-024-44854-7 (PMC10796634; doi:10.1038/s41467-024-44854-7)
Supplement: Supplementary file 3 — Reporting Summary [file 41467_2024_44854_MOESM3_ESM.pdf]

nature portfolio

Corresponding author(s): Jian-Bo Wan

Last updated by author(s): Dec 16, 2023

Reporting Summary

Nature Portfolio wishes to improve the reproducibility of the work that we publish. This form provides structure for consistency and transparency in reporting. For further information on Nature Portfolio policies, see our Editorial Policies and the Editorial Policy Checklist.

Statistics

For all statistical analyses, confirm that the following items are present in the figure legend, table legend, main text, or Methods section.

n/a	Confirmed	
☐	☒	The exact sample size (*n*) for each experimental group/condition, given as a discrete number and unit of measurement
☐	☒	A statement on whether measurements were taken from distinct samples or whether the same sample was measured repeatedly
☐	☒	The statistical test(s) used AND whether they are one- or two-sided<br>*Only common tests should be described solely by name; describe more complex techniques in the Methods section.*
☐	☒	A description of all covariates tested
☐	☒	A description of any assumptions or corrections, such as tests of normality and adjustment for multiple comparisons
☐	☒	A full description of the statistical parameters including central tendency (e.g. means) or other basic estimates (e.g. regression coefficient) AND variation (e.g. standard deviation) or associated estimates of uncertainty (e.g. confidence intervals)
☒	☐	For null hypothesis testing, the test statistic (e.g. *F*, *t*, *r*) with confidence intervals, effect sizes, degrees of freedom and *P* value noted<br>*Give P values as exact values whenever suitable.*
☒	☐	For Bayesian analysis, information on the choice of priors and Markov chain Monte Carlo settings
☒	☐	For hierarchical and complex designs, identification of the appropriate level for tests and full reporting of outcomes
☒	☐	Estimates of effect sizes (e.g. Cohen's *d*, Pearson's *r*), indicating how they were calculated

Our web collection on statistics for biologists contains articles on many of the points above.

Software and code

Policy information about availability of computer code

Data collection	LC-UV analysis: Agilent 1290 Infinity II UPLC system; Agilent 1260 Series HPLC system;<br>Enzyme identification: Nano-LC-LTQ Orbitrap Velos Pro MS (ThermoFisher Scientific);<br>Substrate selectivity tests: DIONEX Ultimate 3000 UHPLC system coupled with an LTQ-Orbitrap XL mass spectrometer<br>Identification of hydrolysis products: Avance 500 MHz NMR spectrometer (BrukerCo., Karlsruhe, Germany)
Data analysis	LC-UV: OpenLAB CDS ChemStation Edition (Agilent)<br>LC-MS: Xcalibur 2.1 software (Thermo Fisher Scientific)<br>NMR: TopSpin version 3.2 pl 6 (Bruker Biospin)<br>Western blot: Fuji distribution of Image J (version 2.0.0)<br>Statistics: Prism 8 (Graphpad Software Inc, version 8.4.3)

For manuscripts utilizing custom algorithms or software that are central to the research but not yet described in published literature, software must be made available to editors and reviewers. We strongly encourage code deposition in a community repository (e.g. GitHub). See the Nature Portfolio guidelines for submitting code & software for further information.

Data

Policy information about availability of data

All manuscripts must include a data availability statement. This statement should provide the following information, where applicable:

- Accession codes, unique identifiers, or web links for publicly available datasets
- A description of any restrictions on data availability
- For clinical datasets or third party data, please ensure that the statement adheres to our policy

The sequence data for glucosidase genes generated in this study have been deposited in the National Center for Biotechnology Information (NCBI) GenBank Sequence Read Archive under accession code OQ813637 (PnGH1), OQ813638 (PgGH1), OQ813639 (PgGH2), OQ813640 (PqGH1). The sequence data for fungus used in this study are available in the NCBI database under accession code OP120946 [https://www.ncbi.nlm.nih.gov/search/all/?term=ON081646] (M. acerina), OP120959 [https://www.ncbi.nlm.nih.gov/search/all/?term=OP120959] (A. panax), ON081646 [https://www.ncbi.nlm.nih.gov/search/all/?term=ON081646+] (F. oxysporum), and MZ433189 [https://www.ncbi.nlm.nih.gov/search/all/?term=MZ433189+] (C. orchidophilum). Source data are provided with this paper.

Research involving human participants, their data, or biological material

Policy information about studies with human participants or human data. See also policy information about sex, gender (identity/presentation), and sexual orientation and race, ethnicity and racism.

Reporting on sex and gender	n/a
Reporting on race, ethnicity, or other socially relevant groupings	n/a
Population characteristics	n/a
Recruitment	n/a
Ethics oversight	n/a

Note that full information on the approval of the study protocol must also be provided in the manuscript.

Field-specific reporting

Please select the one below that is the best fit for your research. If you are not sure, read the appropriate sections before making your selection.

☒ Life sciences ☐ Behavioural & social sciences ☐ Ecological, evolutionary & environmental sciences

For a reference copy of the document with all sections, see nature.com/documents/nr-reporting-summary-flat.pdf

Life sciences study design

All studies must disclose on these points even when the disclosure is negative.

Sample size	Sample sizes were indicated in the each figure legend, and chosen as so to provide sufficient power for statistical comparison where appropriate.
Data exclusions	Data data have not been excluded.
Replication	Experiments were independently repeated as indicated in the figure legends and could have been successfully replicated. For western blot, three biological replicates were taken to verify the reproducibility of the experimental findings.
Randomization	Plants were allocated randomly among replicates and treatments
Blinding	Blinding not used due to the randomization of plans among groups.

Reporting for specific materials, systems and methods

We require information from authors about some types of materials, experimental systems and methods used in many studies. Here, indicate whether each material, system or method listed is relevant to your study. If you are not sure if a list item applies to your research, read the appropriate section before selecting a response.

Materials & experimental systems

n/a	Involved in the study	
☒	☐	Antibodies
☒	☐	Eukaryotic cell lines
☒	☐	Palaeontology and archaeology
☒	☐	Animals and other organisms
☒	☐	Clinical data
☒	☐	Dual use research of concern
☐	☒	Plants

Methods

n/a	Involved in the study	
☒	☐	ChIP-seq
☒	☐	Flow cytometry
☒	☐	MRI-based neuroimaging
